# Peptide Electrostatic Modulation Directs Human Neural Cell Fate

**DOI:** 10.1002/advs.202507946

**Published:** 2025-09-29

**Authors:** Laura Perez‐Chirinos, Xavier Barceló, M. Gabriella Chiariello, Irene Sanz, Amaia Iturrospe, Arantxa Arbe, J. Alberto Ortega, Siewert J. Marrink, Aitziber L. Cortajarena, Zaida Álvarez, Ivan R. Sasselli

**Affiliations:** ^1^ Center for Cooperative Research in Biomaterials (CIC biomaGUNE) Basque Research and Technology Alliance (BRTA) Paseo de Miramón 194 Donostia‐San Sebastián 20014 Spain; ^2^ Biomaterials for Neural Regeneration Group Institute for Bioengineering of Catalonia (IBEC) The Barcelona Institute of Science and Technology (BIST) Barcelona 08028 Spain; ^3^ Zernike Institute for Advanced Materials University of Groningen Nijenborgh 7 Groningen 9747AG Netherlands; ^4^ Centro de Física de Materiales (CFM‐MPC) CSIC‐UPV/EHU Paseo Manuel de Lardizabal 5 Donostia‐San Sebastián 20018 Spain; ^5^ Department of Pathology and Experimental Therapeutics Institute of Neurosciences University of Barcelona L'Hospitalet de Llobregat 08035 Spain; ^6^ Institut d'Investigació Biomèdica de Bellvitge (IDIBELL) L'Hospitalet del Llobregat 08908 Spain; ^7^ IKERBASQUE Basque Foundation for Science Plaza Euskadi 5 Bilbao 48009 Spain; ^8^ CIBER en Bioingeniería, Biomateriales y Nanomedicina CIBER‐BBN Madrid 28029 Spain; ^9^ Center for Regenerative Medicine Northwestern University Chicago IL 60611 USA

**Keywords:** charge screening, human neural progenitor cells, membrane, molecular dynamics, proteomics, self‐assemblies, supramolecular structures

## Abstract

Supramolecular self‐assembled systems have emerged as versatile platforms for engineering biomimetic environments that precisely regulate cellular behavior. These materials have tunable properties such as stiffness, hydrophobicity, and molecular composition, allowing for customization of their structure and function. Despite significant advances, the specific role of electrostatic properties in modulating cellular responses within supramolecular assemblies remains poorly understood. Here, a peptide library with diverse electrostatic profiles is designed to systematically investigate their influence on the bioactivity of supramolecular assemblies for neural regeneration. Combining computational and experimental methods, the self‐assembly conditions of these peptides are optimized to create stable, biologically relevant architectures. Using human neural progenitor cell (hNPC) cultures, it is demonstrated that negatively charged environments enhance cell survival and promote neuronal differentiation. Specifically, high negative charges activate critical signaling pathways, including the mitogen‐activated protein kinase (MAPK) cascade and cell adhesion mechanisms, leading to neuronal lineage commitment. This study establishes a novel framework for the design of supramolecular systems, offering an unprecedented ability to analyze specific parameters in cell behavior. By achieving control beyond conventional biomaterials, this work provides valuable insights into the complex interplay of biophysical and biochemical cues in the native neural microenvironment, with implications for regenerative medicine and biomaterial design.

## Graphical abstract

This study explores the effect of charge on the bioactivity of peptide‐based supramolecular materials. The design of a peptide library with varying charges that self‐assemble into supramolecular fibers is presented, demonstrating how the surface charge of these fibers, used as artificial extracellular matrices, influences the fate of human neural progenitor cells.

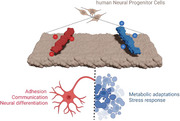

## 1 Introduction

Molecular self‐assembly is a natural process driven by non‐covalent interactions, including hydrophobic interactions, π–π stacking, electrostatic interactions, hydrogen bonding, and van der Waals forces,^[^ ^1^ ^]^ which enables the formation of highly ordered supramolecular structures, such as lipid membranes and virus capsids. Inspired by this self‐assembly process, supramolecular peptide assemblies have attracted considerable attention in biomedicine and bioelectronics in recent years.^[^ ^2^, ^3^, ^4^ ^]^ Composed of amino acids, these peptides exhibit remarkable biocompatibility and bioactivity, along with the unique ability to spontaneously form highly ordered and stable architectures from simple molecular units in solution. The self‐assembly process provides a straightforward way to achieve tunable functionality by exploiting the inherent versatility of peptides by designing and modifying their sequences and structures for specific applications.

The use of supramolecular materials to mimic extracellular matrices (ECMs) and incorporate specific bioactive epitopes has shown exceptional promise for regenerative medicine.^[^ ^5^, ^6^, ^7^ ^]^ Their high tunability allows for precise control over mechanical, physical, and biological properties, making them ideal candidates for designing customized artificial ECMs tailored to various biomedical applications, including neural and musculoskeletal tissue engineering.^[^ ^8^, ^9^, ^10^ ^]^ By providing biomimetic environments that actively modulate cell behavior, these systems open new avenues for functional tissue repair. Recent studies have shown that strategic peptide sequence modifications within amphiphilic monomers can enhance molecular motion within fibrils, leading to dramatic increases in bioactivity and functional recovery in vivo following severe spinal cord injury in a mouse model.^[^ ^11^ ^]^ Furthermore, when incorporating laminin‐derived IKVAV sequences, these supramolecular assemblies significantly enhanced the functional maturation of human induced pluripotent stem cell (iPSC)‐derived motor neurons in vitro.^[^ ^12^ ^]^ Collectively, these findings position supramolecular materials as highly adaptable bioengineering tools, with the potential to revolutionize both in vitro disease modeling and in vivo regenerative therapies, bridging the gap between biomaterial innovation and clinical translation.

Understanding how the properties of supramolecular structures affect their function is crucial for the development of materials with tailored properties. The relationship between the chemical properties of peptides and the morphology and function of the resulting supramolecular structures has been extensively studied by modifying various parameters, including peptide sequence, chirality, charge density, hydrophobicity, and environmental factors such as pH and salt concentration.^[^ ^13^, ^14^, ^15^, ^16^, ^17^, ^18^ ^]^ A key factor that plays a fundamental role in determining the morphology of supramolecular structures is the net charge of the peptide.^[^ ^18^ ^]^ Changes in charge sign and density affect aggregation tendencies, modulate fiber length, and influence fiber bundling through the electrostatic interactions between charged residues.^[^ ^13^ ^]^ In addition, the surface exposure of positive and negative charges has been extensively studied in traditional polymers due to its biological significance. In general, positively charged materials have shown improved bioactivity, playing a critical role in modulating the interaction of polymers with cells.^[^ ^19^ ^]^ In supramolecular systems, however, the role of charge is more complex, as it involves a delicate balance between internal interactions within the assembly and interactions with the cell membrane. While this property has not been extensively explored in supramolecular systems, a recent study demonstrated that neutral and negatively charged self‐assembling peptide hydrogels exhibited higher biological activity in a spheroid model of a human liver cancer cell line than positively charged peptide hydrogels.^[^ ^20^ ^]^ Conversely, supramolecular systems with positive charges tend to show some degree of toxicity.^[^ ^21^ ^]^ Moreover, charged biomaterials provide unique advantages in polarized tissues such as neural and cardiac tissue, supporting improved functionality and compatibility with native tissue characteristics.^[^ ^22^, ^23^, ^24^ ^]^ These findings underscore the importance of surface charge in designing supramolecular biomaterials for optimal cell signaling and interaction. However, the full extent of this parameter's influence remains to be comprehensively investigated.

Therefore, in this work, we developed a library of self‐assembling peptides with different charge densities to investigate the influence of charge on the bioactivity of the resulting fibers, using them as artificial ECM. The self‐assembly of these peptides into supramolecular structures was studied using both computational and experimental approaches, followed by an analysis of their bioactivity. After computationally evaluating optimal conditions and confirming the self‐assembly ability of the peptides using coarse‐grained molecular dynamics (CGMD) simulations based on the Martini model,^[^ ^25^ ^]^ we applied these conditions experimentally to generate supramolecular fibers. Subsequent CGMD simulations of the interactions between these fibers and the neural plasma membrane revealed charge‐dependent contact patterns between the membrane and the fibers. Experimental results showed that negatively charged fibers enhanced the survival and differentiation of human neural progenitor cells (hNPCs) by activating specific cell signaling pathways. These findings not only provide insight into the cytotoxicity of naturally occurring peptide‐based assemblies but also describe and support the rational design of bioactive materials for regenerative medicine applications.

## 2 Results and Discussion

### 2.1 Design of a Peptide Library and Computational Study to Evaluate Their Self‐Assembling Properties

**Figure 1:**
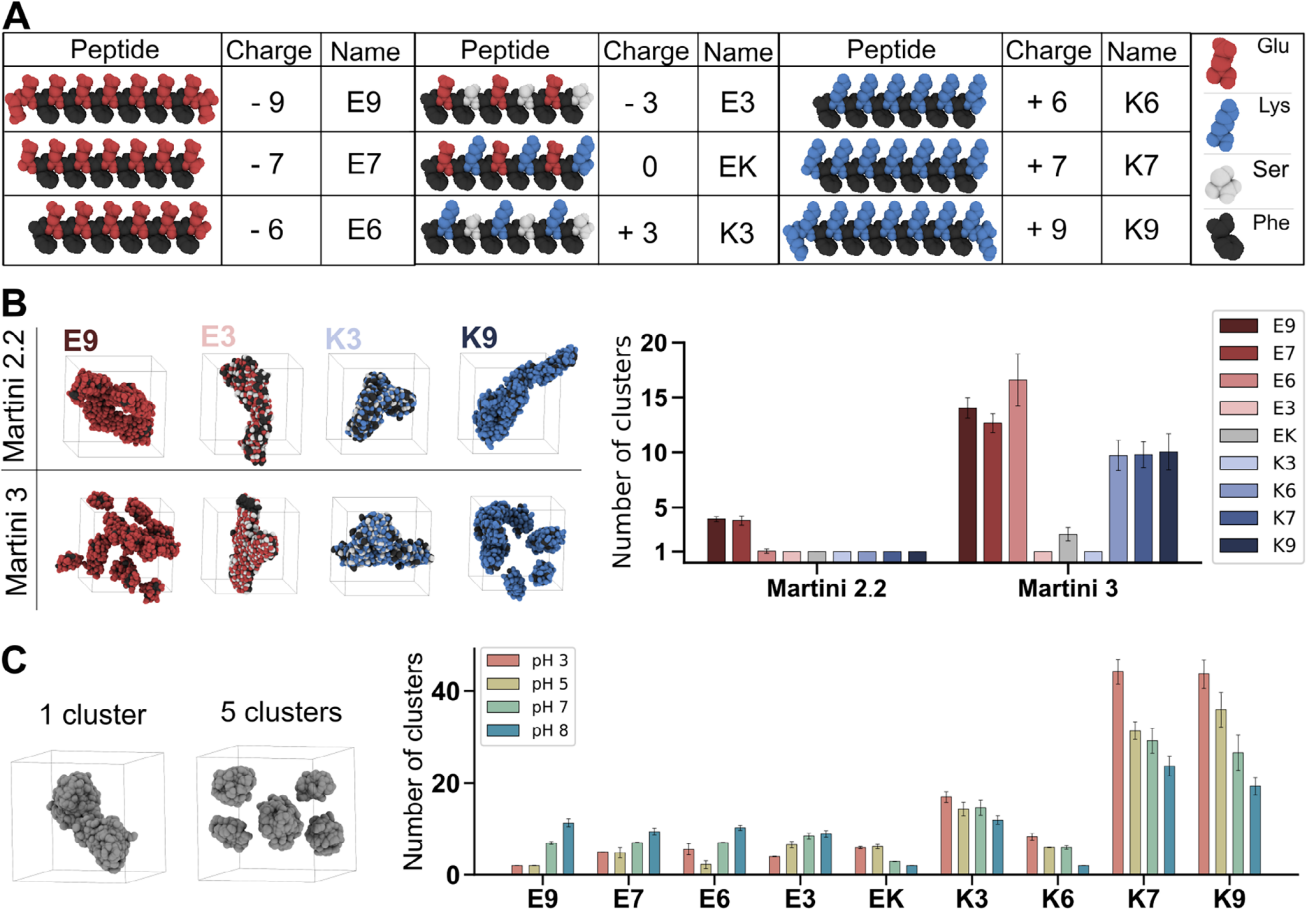
Peptide library and computational study of amphiphilic peptides. A) Table showing the designed peptides, their corresponding charges from −9 to +9, and their assigned names. Glutamates are highlighted in red, lysines in blue, serines in white, and phenylalanines in black. B) Last simulation frame for **E9**, **E3**, K3, and **K9** for Martini 2.2 and Martini 3 force fields (left) and averaged number of clusters during the last 0.25 µs of simulations (right). C) Examples for a system with 1 and 5 clusters of the simulations performed with the titratable model at different pH values (left) and the average number of clusters during the last 25 nanoseconds (ns) for all peptides in the library. The results are presented as the mean and standard deviation of the clusters during the last 0.25 µs in panel (B) and the last 25 ns in panel (C) for a single simulation (*n* = 1).

We designed nine amphiphilic peptide sequences, creating a library that spans a range of net charges from −9 to +9. Their design is based on consecutive repeats of aromatic and hydrophilic residues, imparting an amphiphilic nature that promotes their spontaneous self‐assembly into supramolecular structures. Phenylalanine (F) was chosen to trigger aggregation through hydrophobic interactions as the initial step of assembly. Six phenylalanines alternate with hydrophilic residues in the sequence, with the phenylalanines laying on one side of the peptide backbone and the hydrophilic residues on the other. Negative charges are introduced via glutamic acids (E), while lysines (K) provide positive charges. Serines (S) serve as neutral polar residues, used to modulate the net charge when needed. This is achieved by substituting charged residues in alternating positions, ensuring an even distribution of charges and preserving overall polarity. This design promotes the formation of a hydrophobic core surrounded by hydrophilic residues that stabilize the interphase with water. Anisotropic growth to form 1D self‐assembled fibers is driven by π‐stacking of phenylalanine aromatic rings and hydrogen bonding between the peptide backbones. The charge of the six‐repeat dipeptide (F*X*)_6_, where *X* is the polar residue, ranges from −6 to +6 depending on combinations of E, K, or S. To further increase the net charge, additional E or K residues were incorporated at the N‐ and C‐termini of the core structure, extending the range to −9 or +9. This extensive peptide library is shown in **Figure** **1**A, where the negatively charged peptides are **E9** (EEFEFEFEFEFEFEE), **E7** (EFEFEFEFEFEFE), **E6** (FEFEFEFEFEFE), and **E3** (FEFSFEFSFEFS), the positively charged peptides are **K9** (KKFKFKFKFKFKFKK), **K7** (KFKFKFKFKFKFK), **K6** (FKFKFKFKFKFK), and **K3** (FKFSFKFSFKFS), and the neutrally charged peptide, that combines the E and K residues to neutralize the net charge, is called **EK** (FEFKFEFKFEFK).

#### 2.1.1 Martini 2.2 versus Martini 3 to Evaluate Peptide Self‐Assembly

Coarse‐grained molecular dynamics (CGMD) simulations have become a valuable tool for screening short peptide sequences to identify potential candidates for the formation of ordered, self‐assembled supramolecular materials. Inspired by previous examples that used MD simulations to screen peptides based on their self‐assembly capabilities,^[^ ^26^ ^]^ we performed CGMD simulations to evaluate the self‐assembly tendencies and resulting structures of the proposed peptide library (Figure 1B). The Martini 2.2 force field, which has been widely validated for the study of peptide self‐assembly,^[^ ^27^ ^]^ was chosen to predict the behavior of the peptides in our library. In addition, we sought to explore the capabilities of the newer Martini 3, which holds significant promise for applications in the field of biomaterials due to its improved dynamics and flexibility in protein modeling.^[^ ^28^, ^29^ ^]^ Further model optimizations have enabled the Martini 3 model to reproduce the behavior of intrinsically disordered proteins (IDP), suggesting that the new model could be applied to a wider range of cases.^[^ ^30^ ^]^ However, it has not yet been benchmarked for short peptide self‐assembly. Therefore, we performed simulations using both Martini 2.2 and Martini 3 to compare the self‐assembly propensity of the nine designed peptides between the two models.

To quantitatively analyze the formation of assemblies, we measured the average number of clusters—independent aggregates—during the last 0.25 µs of the 1.25 µs simulations.^[^ ^31^ ^]^ Results using Martini 2.2 showed that all peptides predominantly formed a single cluster (Figure 1B, right), indicating the presence of only one large assembly in the simulation box, where all peptides had collapsed (Figure 1B, left; Figure S1A, Supporting Information). In contrast, simulations with Martini 3 showed a significant increase in the number of clusters for peptides with higher charge densities, reflecting the formation of smaller aggregates and, consequently, a lower tendency to self‐assemble (Figure 1B, right).

The improvements in Martini 3 for reproducing protein dynamics have previously been reported to increase solubility, which negatively affects the modeling of self‐assembly of short peptides.^[^ ^29^, ^32^ ^]^ Interestingly, in our case the peptides are longer than those in the previous studies, which used di‐ and tripeptides, and this discrepancy between the Martini 2.2 and Martini 3 arises only for the more soluble peptides. For the more neutral and less soluble peptides (i.e., **E3** and **K3**), simulations with Martini 3 showed a reduced number of clusters compared to the peptides with higher charge density (Figure 1B, right), revealing a morphology closer to what is expected for a supramolecular self‐assembly (Figure 1B, left; Figure S1A, Supporting Information). **E3** and **K3** showed phenylalanine side chains buried in the core of the assembly in Martini 3, whereas in Martini 2.2, a greater number of these hydrophobic side chains are exposed on the surface (Figure 1B, left; Figure S1A, Supporting Information). Although a more detailed structural analysis is needed to confirm this, and there are no self‐assembly indicators for the more soluble peptides (charge > |3|), Martini 3 appears to provide a more realistic molecular arrangement within the assemblies than Martini 2.2. This is because the hydrophobicity of its beads is higher, which may lead to increased non‐specific aggregation.

#### 2.1.2 Constant pH Simulations to Predict Peptide Self‐Assembly with Martini 3

The discrepancy between the two models arises from the behavior of highly charged peptides, which form only small aggregates in Martini 3. Previous studies using CGMD simulations have reported that highly charged peptide derivatives often require partial neutralization of charges to reproduce the experimentally observed fiber formation.^[^ ^18^, ^33^ ^]^ Although these studies used pH values at which acid groups are expected to be deprotonated, it has been shown that the close proximity of charged groups in aggregates can shift their pKa values, resulting in charge neutralization that promotes the formation of higher order assemblies.^[^ ^34^, ^35^ ^]^ These new transitions, defined by apparent pKa values, have been reported experimentally and theoretically to differ by more than 2 pH units from the pKa of the chemical group under dilute conditions.^[^ ^35^, ^36^ ^]^ Thus, it is possible that the greater solubility captured by Martini 3 is a better representation of the behavior of the fully charged peptide, and that charge neutralization may be necessary to induce self‐assembly in peptides with greater solubility. However, this is a complex, sequence‐ and pH‐dependent scenario that requires the use of a constant pH model. The titratable model in the Martini 3 force field allowed us for constant pH simulations to evaluate pH effects, and has been previously validated for small molecules, polymers, lipids, and proteins^[^ ^37^, ^38^ ^]^ but has not yet been used for peptide self‐assembly.

Using the Martini titratable model, the nine peptides were simulated for 100 ns at four different pH values (3, 5, 7, and 8). These simulations are significantly shorter than the ones described in Section 2.1.1, motivated by the short equilibration times observed in the latter (Figure S1B, Supporting Information) and the higher computational cost of the titratable model. While these results do not guarantee full equilibration in all cases, they are sufficient to demonstrate a clear pH‐dependent self‐assembly behavior in the target sequences. The number of clusters, averaged over the last 25 ns of the simulations, showed a pH‐dependent tendency to aggregate for all peptides. Negatively charged E‐containing peptides showed higher aggregation at lower pH values, whereas positively charged peptides showed increased aggregation at higher pH values (Figure 1C; Figure S2, Supporting Information). This indicates that the peptides tend to have a higher aggregation propensity at a pH close to the respective pKa values of their main charged amino acids. Furthermore, the results of the titratable simulations showed that the number of clusters is consistently higher for **K3**, **K7**, and **K9** at all pH values compared to the other peptides (Figure 1C). This observation may be due to a limitation associated with the pH range of the model. The pKa value of the lysine side chain (10.79)^[^ ^39^ ^]^ exceeds the maximum pH achievable with the model, which is pH 8. At pH > 8, the model becomes numerically unstable, which may contribute to the higher instability observed in simulations of peptides with a greater number of lysines. In addition, the highest pH studied^[^ ^8^ ^]^ is 2 units below the pKa of the K residues, meaning that the simulations were unlikely to fully neutralize the positive charges and reduce the electrostatic repulsion between peptides. This results in a lower aggregation tendency for K‐containing peptides. In contrast, E residues have a pKa value (4.25),^[^ ^39^ ^]^ within the pH range studied. The differences in the side chain characteristics, such as the number of beads (two beads for K and one for E) and hydrophobicity, make direct comparison of absolute aggregation between peptides difficult in this case. However, the framework still allows meaningful comparisons across different pH values for individual peptides. Consistent with this, results from the titratable model suggest that highly charged peptides in the library may require pH conditions closer to the pKa values of their side chains to reduce charge repulsion and achieve optimal self‐assembly behavior.

Taken together, these results highlight the differences between Martini 2.2 and Martini 3 in modeling peptide self‐assembly. Martini 3 showed a reduced propensity of highly charged peptides to self‐assemble, suggesting that such peptides would not be able to self‐assemble into filaments. This observation is consistent with the previously reported increased solubility of short peptides in Martini 3. However, incorporation of the titratable model revealed that peptides with higher charge densities may require pH conditions closer to their pKa to achieve effective aggregation. These results suggest a potential pH‐dependent behavior in the experimental self‐assembly of the peptides in our library.

### 2.2 Experimental Characterization of the Peptide Library

**Figure 2:**
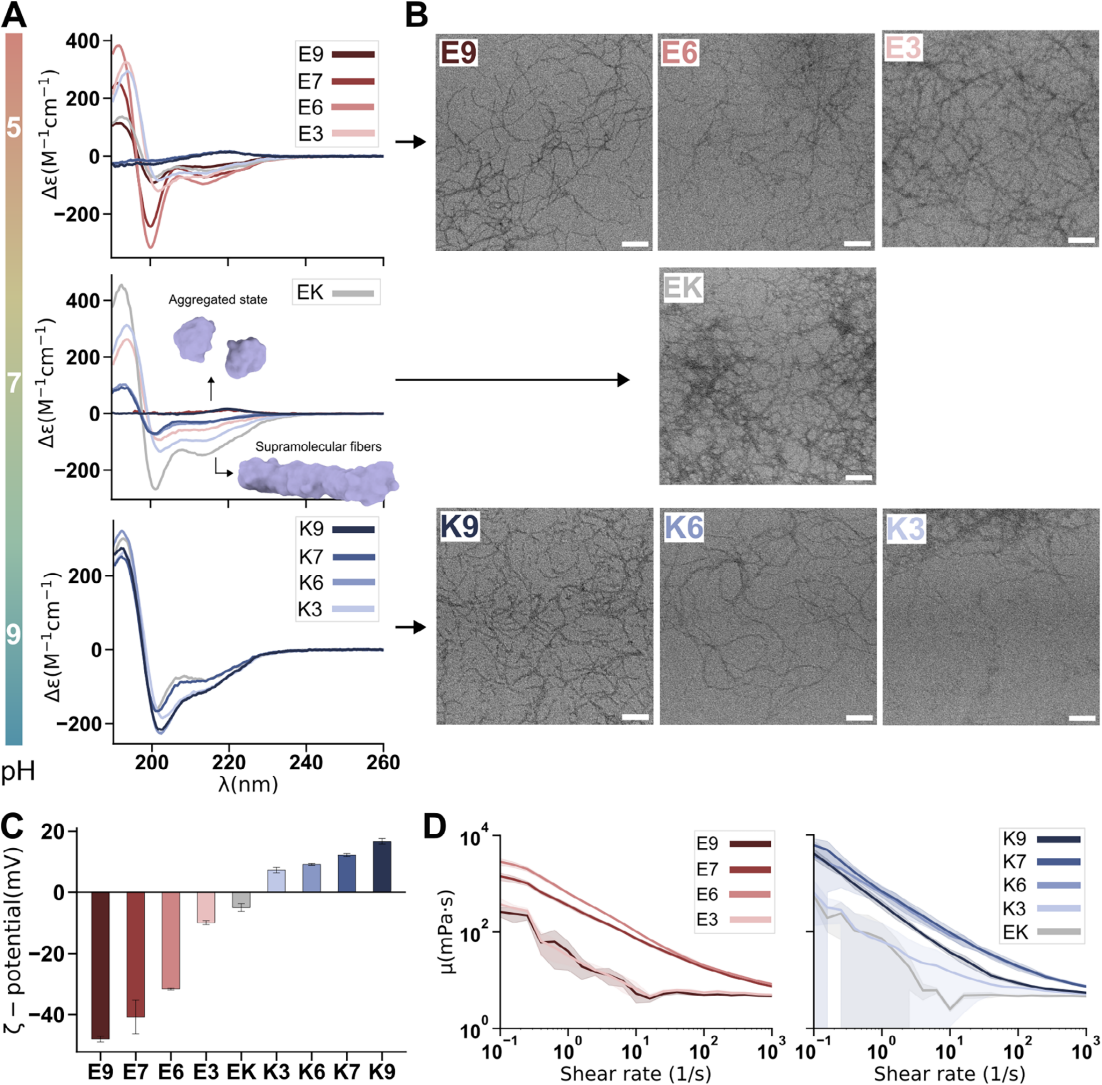
Experimental characterization of the amphiphilic peptide library. A) CD spectra of the peptides at pH 5 (top), pH 7 (middle), and pH 9 (bottom). B) TEM images of supramolecular fibers at their optimal pH conditions: pH 5 for **E9**, **E6**, and **E3** (top); pH 7 for **EK** (middle); and pH 9 for **K9**, **K6**, and **K3** (bottom). Scale bars: 100 nm. C) Zeta potential characterization of the fibers. D) Viscosity measurements of E fibers (left) and K fibers (right) from the peptide library. In panels (C) and (D), the results are presented as the mean and standard deviation of three different measurements (*n* = 3).

Based on the potential influence of pH on the self‐assembly behavior of the peptides in the library, we experimentally investigated the formation of fibers at three pH values (5, 7, and 9) (**Figure** **2**A). Given the design of our library, the formation of supramolecular structures depends on the peptides arranging themselves in a β‐sheet configuration. By analyzing the formation of these β‐sheet structures using circular dichroism (CD) spectroscopy, we evaluated the self‐assembly process. The CD spectra revealed a β‐sheet secondary structure (negative peak at 215 nm and positive peak at 195 nm) stabilized by π‐stacking (negative peak at 200 nm) of the phenylalanine side chains, at pH 5 for all E‐containing peptides, **EK**, and **K3**. Instead, at pH 7 and 9, the negative **E9**, **E7**, and **E6** exhibited random coil CD patterns, indicating a lack of intermolecular order and thus self‐assembly. At these pH values, electrostatic repulsion between the deprotonated side chains of the glutamic acids likely prevents the assembly. In contrast, the serine residues in **E3** mitigate charge repulsion and allowed fiber formation at pH 7 (Figure 2A). The positively charged **K9**, **K7**, and **K6** did not show secondary structure CD signals at pH 5, suggesting that fibers are not being formed. However, the inclusion of serines, as in **E3**, neutralizes some charge repulsion, allowing **K3** to form supramolecular order even at pH 5. At pH 7, **K9**, **K7**, and **K6** showed secondary structure signals, indicative of self‐assembly, likely due to deprotonation of lysine side chains, which reduces peptide repulsion. Interestingly, at pH 9, further deprotonation of lysine side chains increased peptide affinity and enhanced the contribution of π‐stacking (Figure 2A). Finally, **EK** represents a unique case in that it contains both positively and negatively charged residues, resulting in an overall neutral charge. **EK** forms secondary structure signals at all three pH values, indicating consistent self‐assembly into nanostructures over the pH range studied (Figure 2A). These results demonstrate that the pH‐dependent supramolecular ordering is strongly influenced by peptide sequence. Peptides with higher net charge require pH conditions closer to their pKa values.

We used Fourier transform infrared (FT‐IR) spectroscopy to confirm the formation of secondary structures and to gain further insight into the molecular arrangement at the optimal pH for self‐assembly, i.e., pH 5 for the E‐based peptides, pH 7 for **EK**, and pH 9 for the K‐based peptides. The FT‐IR peaks at 1615–1620 cm^−1^ represent the hydrogen bonding of the β‐sheet secondary structures, while the small peaks at 1670–1700 cm^−1^ (in some cases masked by a larger TFA peak at 1673 cm^−1^) represent the antiparallel coupling between the strands (Figure S3A, Supporting Information), confirming fiber formation with a β‐sheet structure as observed by CD and determining an antiparallel arrangement of the sheets. In addition, transmission electron microscopy (TEM) was used to confirm the fiber formation suggested by the intermolecular order revealed by both spectroscopic techniques. TEM micrographs confirmed the formation of 1D supramolecular fibers with similar morphology under the studied conditions (Figure 2B; Figure S3B, Supporting Information). Interestingly, TEM images revealed that **EK** and **K3** peptides form bundled fiber structures, suggesting an important role for lysine residues in bundling. These results are consistent with the conformations observed in the titratable simulations, where **K6** at pH 8 also showed strong interactions between lysines, inverting the peptide conformation, partially burying the more hydrophilic lysine side chains in the core and exposing the more hydrophobic phenylalanines (Figure S2A, Supporting Information). Lysines showed a greater tendency to interact, when neutral, compared to glutamic acids. This behavior could be attributed to the increased length of the lysine side chain, which provides greater flexibility and hydrophobicity compared to glutamic acid, as reflected by its higher octanol‐water partition coefficient logP_Lys_ = −2.04 versus logP_Glu_ = −2.64.^[^ ^40^, ^41^ ^]^ In addition, the higher charge delocalization in the amine group of the lysines reduces the repulsion between their side chains. This reduced repulsion, combined with the increased flexibility and hydrophobicity of the lysine side chain, may explain their strong contribution to peptide aggregation under these conditions. For **EK**, titratable simulations also showed an inverted conformation compared to that was expected at pH 7 and 8, with the hydrophilic groups buried in the core and the phenylalanines exposed (Figure S2A, Supporting Information). The bundling effect in **EK** is further enhanced by electrostatic interactions between the complementary charges of lysines and glutamic acids, suggesting that **EK** fibers are formed by a combination of electrostatic and hydrophobic effects.

To evaluate the surface charge of the supramolecular fiber structures and the potential differences in their mechanical properties, we investigated the zeta potential and the rheological behavior of the fibers. Zeta potential measurements are challenging for these types of supramolecular structures because it estimates the surface charge based on a model that describes the mobility of spherical particles under an electric field. Since our fibers clearly deviate from spherical morphology, vary in length, and exhibit fiber–fiber interactions, these measurements may not be completely accurate. To address this, we mitigated some of these limitations by breaking the long fibers into smaller, more diffuse ones, allowing us to establish a qualitative scale for comparing samples prepared under the same conditions. The zeta potential results confirmed the expected trend in the charge based on the net charge of the designed peptide library (Figure 2C). Regarding the rheological experiments, the analysis showed that all supramolecular materials responded similarly to mechanical shear in the range of 0.1 to 1000 s^−1^ with a non‐zero slope, indicating a pronounced shear thinning behavior (Figure 2D). This suggests that the mechanical properties of the resulting material are primarily determined by the presence of long fibers, given the consistency across different peptides in the library, rather than by charge density and the resulting inter‐filament interactions. The fact that peptides with different charge densities exhibit highly similar rheological profiles underlines the robustness of the library design, which—when combined with the consistent formation of similar structures with antiparallel β‐sheet order—enables the systematic investigation of charge‐specific effects decoupled from other potential contributors.

### 2.3 Computational and Experimental Analysis of Fiber–Neural Cell Interactions

**Figure 3:**
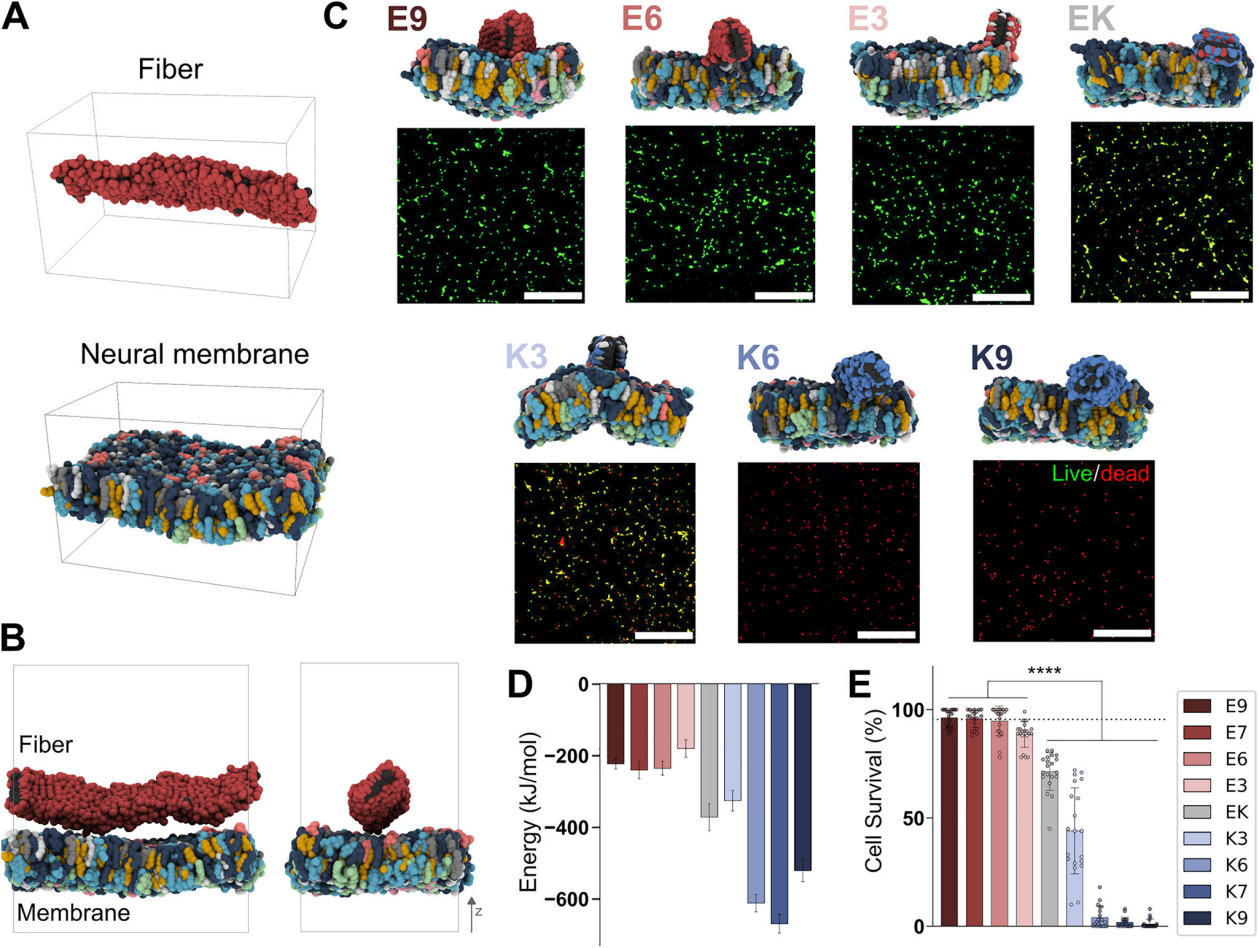
Fiber‐neural membrane interaction. A) Final simulation frame (1.25 µs) of the fiber and the PM, showing the inner and outer leaflets of the neural membrane. B) Initial fiber‐PM configuration with lateral and side views using **E9** as a representative system. The fiber is placed at the top of the outer leaflet of the PM. C) Final simulation frames for all fiber‐PM systems (top). Confocal micrographs of hNPCs treated with different peptide fibers. Calcein (green) stains live cells, and propidium iodide (red) stains dead cells. Scale bar: 100 µm (bottom). D) Average sum of LJ and Coulombic energies (normalized by charged residues) during the last 0.25 µs of simulations in (C). The data presented consists on the mean and standard deviation during the last 0.25 µs for a single simulation (*n* = 1). E) Percentage of hNPC viability after treatment with the peptide library fibers. The dashed line indicates the average cell survival percentage in non‐treated control condition. Data were obtained from at least three independent differentiations. All values are presented as mean ± SEM. Statistical significance was assessed using one‐way ANOVA followed by Tukey's post hoc test, with *p* < 0.05 considered significant. *p < 0.05; **p < 0.01; ***p < 0.001; ****p < 0.0001.

To investigate the influence of charge on the bioactivity of the materials, we first analyzed the interactions between fibers from the peptide library and neural cell membranes using CGMD simulations. Neural cells were chosen because of the unique lipid composition of their membranes, which are characterized by high levels of cholesterol and unsaturated lipid tails.^[^ ^42^ ^]^ This lipid composition is critical for mediating interactions with proteins and other biomolecules in their environment, which are essential for critical neuronal processes, cellular signaling, adhesion, and response to external stimuli. Given the highly charged nature of the membrane surface, we hypothesized that the varying charges in our proposed material library could influence and potentially tune the membrane–fiber interaction. To perform the fiber‐membrane simulations, a 1D infinitely interconnected fiber model based on experimental observations was built and equilibrated, while for the membrane we used a neural plasma membrane (PM) model previously reported by Ingólfsson et al.^[^ ^43^ ^]^ (**Figures** **3**A, and S4A,B, Supporting Information). This model included 58 different lipids to represent an idealized composition of the human brain PM, distributed in different ratios on the inner and outer leaflets (Table S1; Figure S4B, Supporting Information).^[^ ^43^ ^]^ We used the well‐validated Martini 2.2 version because Martini 3 presented challenges in reproducing fiber stability, which could have compromised the reliability of the results. Moreover, the membrane model in question had already been validated using version 2.2. The fiber/membrane systems were constructed by placing each of the nine fibers on top of the outer PM leaflet at a center‐to‐center distance of 7 nm and tilting them 45° in the *y*‐direction to prevent biased interactions between the fiber sides and the membrane (Figure 3B; Figure S4C, Supporting Information). After 1.25 µs of simulation (Figure 3C, top; Figure S4D, Supporting Information), we evaluated the interaction strength by calculating the sum of the Lennard–Jones (LJ) and Coulombic energy contributions between the PM lipids and the charged residues of the fibers as a proxy for the peptide‐membrane binding strength (Figure 3D).

Our results showed that fibers containing lysines have higher interaction energies with the PM compared to negatively charged fibers (Figure 3D). The outer leaflet of the membrane has 445 positive and 473 negative charges per nm^2^, resulting in an overall negative net charge of −28. This explains the stronger interaction between the membranes and positively charged materials, a relationship that has been widely discussed in the literature.^[^ ^19^ ^]^ Interestingly, the negatively charged fibers also exhibited an attractive interaction with the PM, contrary to the expected repulsion between two negatively charged systems. This attraction may be due to the presence of positive charges on the membrane, which may rearrange to facilitate some degree of interaction with the negatively charged fibers. Therefore, these results suggest that all fibers experience electrostatic interactions with the PM, with the positively charged fibers having stronger interactions with the membranes than the negatively charged fibers.

To experimentally validate the computational results and assess the impact of charge on neural cell behavior, we selected human induced pluripotent stem cell‐derived neural progenitor cells (hNPCs). hNPCs were chosen due to their ability to differentiate into various neural cell types, including astrocytes and neurons, making them an ideal model for evaluating how charge‐modulated supramolecular matrices influence neural cell viability, survival, and differentiation.^[^ ^11^, ^44^, ^45^ ^]^ To investigate the effects of amphiphilic peptide fibers on hNPCs, cells were treated with the library of peptides in solutions for seven days in vitro (see Section 4). A live/dead assay using Calcein and propidium iodide (PI) revealed significant differences in cell viability between negatively and positively charged fibers (Figure 3C, bottom; Figure S4D, Supporting Information). Negatively charged fibers, which contain glutamic acid residues and have net charges of zero or below, maintained cell survival (Calcein‐positive cells) at levels comparable to the non‐treated control, demonstrating their biocompatibility (Ctrl = 95.5 ± 4%, E9 = 96.2 ± 4%, E7 = 95.9 ± 4%, E6 = 94.8 ± 6%, E3 = 88.5 ± 5%, and **EK** = 71.4 ± 8%). In contrast, positively charged fibers caused a significant increase in cell death, with the percentage of dead cells correlating with the number of positive charges (PI‐positive cells, K9 = 1.1 ± 2%, K7 = 1.8 ± 2%, K6 = 4.1 ± 5%, K3 = 44 ± 19%) after 1 week in vitro (Figure 3C, bottom; Figure 3E; Figure S4D,E, Supporting Information). Western blot analysis further confirmed that hNPCs treated with positively charged and neutral amphiphilic peptide fibers exhibited elevated levels of cleaved CASPASE‐3 (cCASP3), indicating increased apoptotic activity compared to cells treated with negatively charged fibers. This increase in apoptosis correlated with a marked downregulation of INTEGRIN β1 (ITGB1), suggesting cell detachment and, consequently, reduced viability (Figure S4E, Supporting Information). To ensure that the fibers remained structurally stable in cell culture conditions and did not affect the survival of cells, we performed small‐angle X‐ray scattering (SAXS) (Figure S5, Table S2, Supporting Information). Despite increased noise and reduced sensitivity from the complex cell culture media, the SAXS patterns at pH 5 remained well described by a cylinder model, consistent with TEM observations of fibers (Figure 2B; Figure S3B, Supporting Information). At physiological pH, rather than disassembling, the fibers tended to bundle due to the high salt concentration, as indicated by deviations at lower *Q* values. This confirms the stability of the peptide nanostructures in a cellular environment, supporting their potential for biological applications. Taken together, these findings underscore the critical role of charge‐dependent interactions in modulating fiber bioactivity. Positively charged fibers disrupt membrane integrity, consistent with the stronger interactions observed in molecular dynamics simulations, whereas negatively charged fibers exhibit weaker interactions, preserving cell viability.

### 2.4 Electrostatic Charge of Supramolecular Fibers Modulates Specification of Human Neural Progenitor Cells (hNPCs)

**Figure 4:**
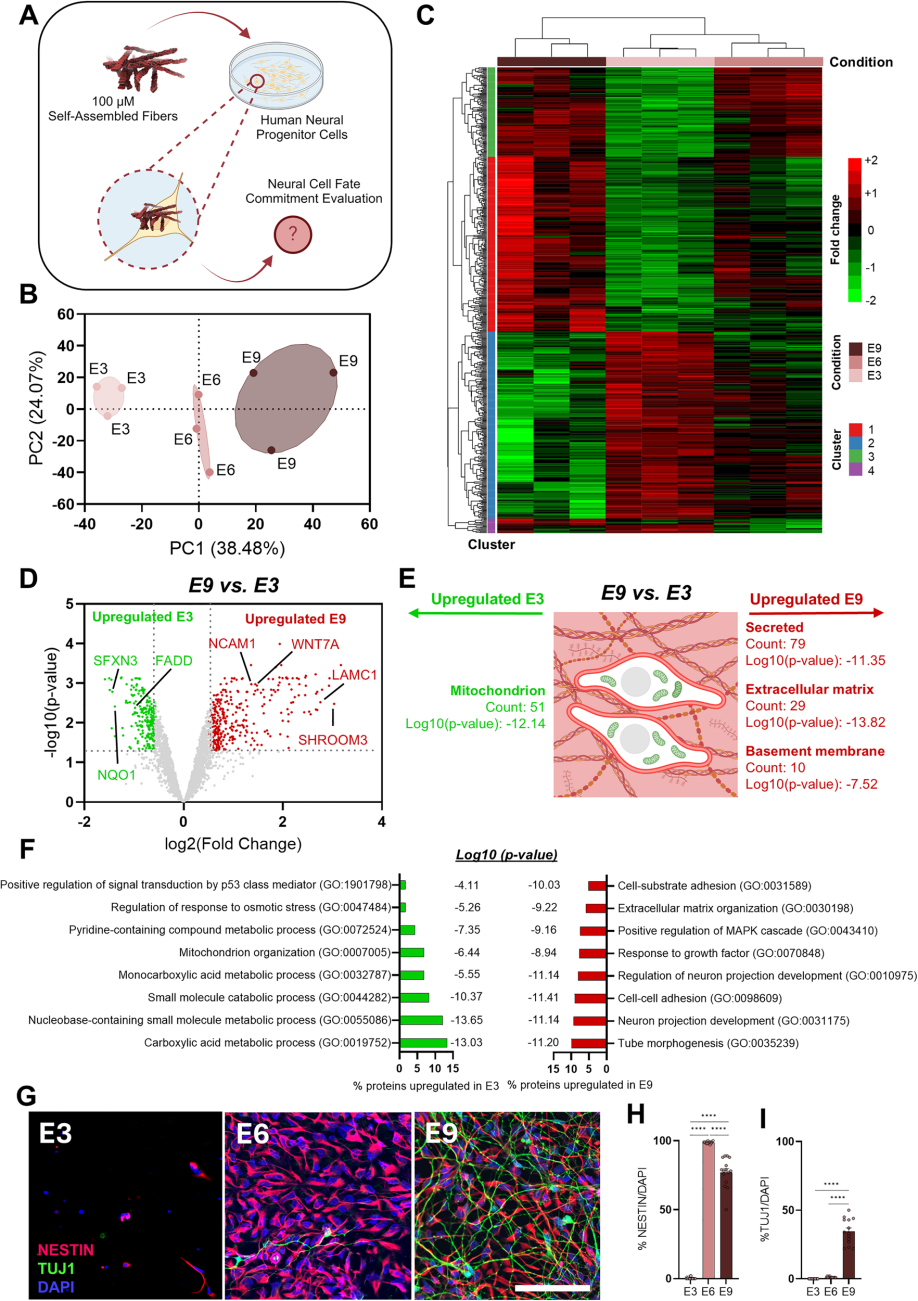
Electrostatic charge variation in self‐assembled fibers drives differential neural cell fate in hNPCs. A) Schematic of the experimental workflow for cell culture and proteomic analyses. B) PCA of proteomic data (3690 proteins; *n* = 3) showing distinct clustering of experimental groups (**E3**, **E6**, **E9**) and controls. C) Hierarchical clustering heatmap of differentially abundant proteins, identifying four distinct protein clusters. D) Volcano plot showing fold‐change (*x*‐axis) versus significance (−log10 *p*‐value, *y*‐axis) for protein expression in hNPCs treated with **E3** or **E9** fibers. Upregulated proteins (|FC| > 1.5 and *p* < 0.05) are marked in red (**E9**) or green (**E3**), with key proteins labeled. E) Schematic representation of the subcellular localization of significantly upregulated proteins (*p* < 0.05) in **E3** (green) and **E9** (red) treatment groups. F) Gene Ontology (GO) analysis highlighting enriched biological processes, with *p*‐values and percentages of total protein content displayed. G) Representative immunocytochemistry (ICC) confocal images after 2 weeks of culture showing NESTIN (red), TUJ1 (green), and DAPI (blue). Scale bar: 100 µm. H) Bar graph showing the percentage of NESTIN‐positive cells normalized to DAPI. I) Bar graph showing the percentage of TUJ1‐positive cells normalized to DAPI. Data were obtained from at least three independent differentiations. All values are presented as mean ± SEM. Statistical significance was assessed using one‐way ANOVA followed by Tukey's post hoc test, with *p* < 0.05 considered significant. *p < 0.05; **p < 0.01; ***p < 0.001; ****p < 0.0001.

We next examined whether the density of negative charges within the supramolecular fibers influenced hNPC fate. Since positively charged fibers induced significant cell death, we focused these experiments exclusively on negatively charged fibers (**E3**, **E6**, and **E9**) (**Figure** **4**A). To comprehensively and objectively assess how supramolecular fiber treatments affect hNPC behavior, we performed a proteomic analysis after one week of cell culture. This analysis quantified a total of 3690 proteins and provided important insights into the cellular response. Principal Component Analysis (PCA) of protein abundance data revealed distinct clustering of each experimental group (**E9**, **E6**, and **E3**), with a clear separation from the non‐treated control (CTRL) (Figure 4B; Figure S6A, Supporting Information). These distinct proteomic profiles suggest that the peptide‐based supramolecular fibers elicit specific cellular responses driven by charge density variation. A hierarchical clustering heatmap of differentially expressed proteins further confirmed this trend, showing a clear separation between treated groups (**E9**, **E6**, and **E3**) and the non‐treated control (CTRL), as well as a charge‐dependent pattern among the treated groups (Figure 4C; Figure S6B, Supporting Information).

Four protein clusters were identified based on differential abundance profiles, revealing multiple significant differences between **E9**, **E6**, and **E3** (Figure 4D; Figures S7A and S8A, Supporting Information). Proteins primarily associated with basement membrane (count: 10, Log10(*p*‐value): −7.52), ECM (count: 29, Log10(*p*‐value): −13.82), and secreted factors (count: 79, Log10(*p*‐value): −11.35) were significantly upregulated in **E9** compared to **E3** (Figure 4E). Key proteins such as NCAM1, WNT7A, LAMC1, and SHROOM3, which are involved in cell adhesion, communication, and neural differentiation,^[^ ^46^, ^47^, ^48^, ^49^, ^50^ ^]^ were notably upregulated in the **E9** group, suggesting that this condition may provide a more favorable environment for neural development. Similarly, proteins related to ECM (count: 3, Log10(*p*‐value): −1.51) and secreted factors (count: 7, Log10(*p*‐value): −1.80), which contribute to a supportive microenvironment for neural differentiation, were upregulated in **E9** fiber compared to **E6** (Figure S7B, Supporting Information). In contrast, hNPCs treated with **E3** showed upregulated proteins associated with mitochondrial function (51 proteins, Log10(*p*‐value): −12.14), including FADD, SFXN3, and NQO1, which have been previously linked to apoptosis,^[^ ^51^ ^]^ mitochondrial regulation,^[^ ^52^ ^]^ and oxidative stress response^[^ ^53^ ^]^ (Figure 4E; Figure S8B, Supporting Information).

We further investigated whether common functional terms could be identified among the upregulated proteins in each treatment group. Gene Ontology (GO) analysis revealed significant enrichment of proteins associated with “cell‐cell adhesion,” “tube morphogenesis,” “regulation of neuron projection development,” “extracellular matrix organization,” “cell‐substrate adhesion,” and “positive regulation of the MAPK cascade,” as well as “response to growth factors” in hNPCs treated with **E9** compared to those treated with **E3** fibers (Figure 4F). Similarly, the terms “supramolecular fiber organization,” and “maintenance of location in cell” were enriched in the **E9** group compared to **E6** suggesting a stronger structural role of these fibers in stabilizing cell adhesion and spatial organization within the neural microenvironment (Figure S7C, Supporting Information).

In contrast, proteins associated with “metabolic adaptations” and “stress responses” were predominantly upregulated in the **E3** group compared to **E9** (Figure 4F; Figure S8C, Supporting Information). These findings underscore the critical influence of the electrostatic charge of supramolecular fibers, particularly **E9**, on the functional profiles of hNPCs, emphasizing the role of the physicochemical properties of the cellular microenvironment in driving neural development. To validate these findings, we conducted immunocytochemistry (ICC) in hNPC cultures after two weeks of treatment (Figure 4G). Confocal imaging of hNPCs immunolabeled for the neuronal marker β‐TUBULIN‐III (TUJ1) and the progenitor marker NESTIN, demonstrated notable differences across the supramolecular fiber treatments. Cultures treated with **E3** exhibited a significant reduction in cell number, consistent with their high stress‐related proteomic signature (Figures 3E and 4F; Figure S4E, Supporting Information). The **E6** group displayed a higher proportion of NESTIN‐positive cells, suggesting the maintenance of a larger undifferentiated stem cell population (Figure 4H). In contrast, the **E9** group, exhibited the highest percentage of TUJ1‐positive cells, indicating enhanced neuronal differentiation (Figure 4I; Figure S4E). These findings suggest that negatively charged fibers, particularly **E9**, promoted neuronal differentiation while preserving a subset of undifferentiated progenitor cells. In contrast, treatment with the least negatively charged fibers (**E3**) resulted in a loss of neural progenitors and a shift toward stress‐induced cellular responses, potentially impairing differentiation capacity. While these data strongly support the idea that surface charge influences cell fate, further investigations are necessary to establish explicit links between these molecular‐level observations and the maturation of neurons over time.

The bioactivity exhibited by **E9** fibers likely arises from their ability to mimic the negatively charged ECM environment. The ECM plays a crucial role in neural development, with its composition and electrostatic properties significantly influencing cellular behaviors.^[^ ^54^ ^]^ Notably, the developing ECM is predominantly composed of glycosaminoglycans (GAGs), particularly heparan sulfate and chondroitin sulfate, which confer a strong negative charge.^[^ ^54^, ^55^, ^56^ ^]^ This negatively charged environment regulates key developmental processes, including cell adhesion, proliferation, migration, and morphogenesis, facilitating the dynamic remodeling necessary for successful neurogenesis.^[^ ^57^, ^58^, ^59^ ^]^ As the neural tissue matures, the ECM shifts toward reduced GAG content and an increased presence of structural proteins such as collagen and fibronectin. This results in altered electrostatic interactions that affect cellular behavior differently in the mature nervous system.^[^ ^60^, ^61^ ^]^

Previous studies utilizing electroactive or peptide‐based biomaterials, such as peptide amphiphiles (PAs) and peptides from the RADA family, have demonstrated that altering the net charge of peptides significantly influences cell adhesion, viability, and differentiation.^[^ ^20^, ^62^, ^63^, ^64^, ^65^, ^66^ ^]^ However, these studies typically involved concurrent changes in morphology, mechanical properties, and bioactive ligand presentation, which complicates the precise interpretation of electrostatic effects in isolation.^[^ ^67^ ^]^ Our approach uniquely addresses this limitation by systematically modulating the net charge of peptides without altering their morphology or stiffness, enabling us to assess electrostatic interactions. In line with recent literature,^[^ ^20^ ^]^ our findings highlight the advantages of using negatively charged peptides to enhance neural cell viability and differentiation.

## 3 Conclusions

In this study, we present a strategy for designing peptide‐based artificial ECMs for neural regeneration, providing a framework for understanding the interactions between neural cells and their microenvironment. We developed a peptide library with charge densities ranging from −9 to +9 per peptide and evaluated their self‐assembly tendencies using CGMD simulations. A comparative analysis of Martini 2.2 and Martini 3 models showed that Martini 3 provides a more accurate representation of long peptides while highlighting the need to considering pH conditions near the pKa of the peptides to facilitate aggregation in highly charged systems. These findings allowed us to optimize assembly conditions, leading to the experimental validation of filaments with similar dimensions, intermolecular order, and mechanical properties, but distinct charge profiles—establishing them as an ideal model system for studying the role of charge in bioactivity. Using this peptide library, we investigated the effect of charge density on hNPC bioactivity by employing these materials as synthetic matrices in solution. Our results showed that positively charged fibers induced significant cell death, likely due to their stronger interactions with the cell membrane, whereas negatively charged fibers supported cell survival. Notably, highly negatively charged fibers (**E9**) upregulated proteins associated with neural differentiation, cell adhesion, and cell communication, whereas less negatively charged fibers (**E3**) primarily upregulated stress‐related proteins and mitochondrial activity. These results underscore the critical role of electrostatic charge in regulating cellular responses and highlight the importance of integrating charge considerations into the rational design of biomaterials for neural tissue engineering. Finally, this study demonstrates a multidisciplinary approach to the development of bioactive materials by seamlessly combining computational modeling, experimental characterization, and proteomic analysis. By elucidating the intricate relationship between material properties and cell behavior, our findings provide valuable insights for the rational design of biomaterials with enhanced bioactivity for neural regeneration.

## 4 Experimental Section

### Coarse‐Grained Simulation Procedures for Peptide Self‐Assembly

The initial structures for all the peptides were created in Avogadro^[^ ^68^ ^]^ and transformed into Martini 2.2 CG forcefield^[^ ^27^ ^]^ and Martini 3 CG force field^[^ ^29^ ^]^ representation using martinize.py version 2.6.^[^ ^29^ ^]^ For all the simulations the GROMACS software (version 2022)^[^ ^69^ ^]^ was used. 80 peptides were randomly inserted in a box of 15 nm^3^ to obtain a final peptide concentration of 39.36 mm. This concentration is ≈10 times more than the experimental concentrations used, in agreement with the typical concentrations used for CG simulations to speed up the self‐assembly.^[^ ^26^, ^70^, ^71^ ^]^ The systems were solvated with explicit Martini CG water and the charge was neutralized using Na^+^ and Cl^−^ ions. A first step of minimization was performed using the steepest decent minimization algorithm setting the minimum energy to 2 × 10^3^ kJ mol^−1^ nm^−1^ for 5 × 10^3^ steps. Only for the simulations with Martini 3 a step of equilibration after the minimization was performed for 200 ns using a 10 fs time step at 303 K and isotropic NPT conditions using the Berendsen algorithm at 1 bar pressure.

The production Martini 2.2 and Martini 3 simulations were run for 1.25 µs with a 25 fs time step at NPT ensemble setting the temperature at 303 K by using the v‐rescale thermostat and 1 bar pressure through isotropic coupling using the Berendsen algorithm.

For the constant pH simulations, the titratable Martini model was used,^[^ ^38^ ^]^ with the same setup as described above except the Na^+^ and Cl^−^ ions were removed. The titratable glutamic acid side chain was taken from reference,^[^ ^37^ ^]^ and consists of three beads: a neutral side chain particle (type P2_4.8) accounting for the LJ interactions with all other beads, and two dummy beads (one carrying a charge of −1) regulating the binding of protons. For lysine, as no previous model was available, therefore a new topology was constructed based on the titratable base‐type bead.^[^ ^38^ ^]^ The titratable side chain bead 2 of the lysine was divided also in three beads, a negatively charged side chain particle (type SN6d_10.6R), a neutral dummy bead, and a positively charged dummy particle. The simulation workflow with the titratable model consisted of a minimization step using the steepest decent minimization algorithm, setting the minimum energy to 10 kJ mol^−1^ nm^−1^ for 8 × 10^4^ steps, followed by an equilibration of 10^4^ steps with a 10 fs time step in isotropic NPT conditions at 298.15 K setting the pressure to 1 bar with the Berendsen algorithm. The dynamics were subsequently simulated for 100 ns with a 10 fs time step and isotropic NPT conditions setting the temperature at 298.15 K and the pressure at 1 bar with the Parrinello–Rahman algorithm.

For the analysis in Figure 1B,C the number of clusters was calculated using the *maxclust* function of GROMACS (version 2021)^[^ ^72^ ^]^ and averaged during the last 0.25 µs of the simulation for the Martini 2.2 and Martini 3 comparison, and during the last 25 ns for the titratable simulations. The snapshots represent the systems at the last frame of the simulations. All the visualizations were rendered using Chimera X 1.6.1.^[^ ^73^ ^]^

### Coarse‐Grained Simulation Procedures for the Fiber–Membrane Interaction

The fiber models were built by using the theoretical distance of 5 Å between peptides in the *z*‐axis, based on the reported distance between β‐sheets within self‐assembled structures and adapted to the Martini bead size,^[^ ^74^, ^75^, ^76^ ^]^ an antiparallel disposition between the sheets as observed in the FT‐IR spectra (Figure S3, Supporting Information), and finally, two peptides facing each other to obtain fibers with a hydrophilic surface (Figure S4A, Supporting Information). The final initial model contained 100 peptides, mimicking an infinite 1D fiber within the *z*‐axis, in a 15 × 15 × 25 nm box. The systems were solvated with a 75% content of explicit Martini water, and a 25% content of explicit Martini anti‐freezing water particles and the system's charge were neutralized by using Na^+^ and Cl^−^ ions. The force field used for all the models was Martini 2.2.^[^ ^27^ ^]^ The protocol followed to run the simulations is the same as reported in the section named *Coarse‐grained simulation procedures for peptide self‐assembly* for the simulations using the Martini 2.2 force field.

Regarding the cell membrane, the lipid composition of a brain PM model previously reported by Ingólfsson et al. was used with the Martini 2.2 forcefield,^[^ ^27^, ^43^ ^]^ consisting of 58 different lipids to represent an idealized composition of the human brain PM (Figure S4B, Supporting Information), located in different ratios in the inner/outer leaflets (Table S1, Supporting Information). The bilayer was constructed using the *insane_PM.py* script (file attached in the Supporting Information section) in a 30 × 20 × 15 nm box and solvated with explicit Martini CG water and 100 mm NaCl. The PM membrane was simulated using GROMACS (version 2022).^[^ ^69^ ^]^ The system was minimized using the steepest decent minimization algorithm for 2 × 10^3^ kJ mol^−1^ nm^−1^. After the minimization, two equilibration steps were required, the first with a 10 fs timestep for 5 × 10^4^ steps and the second with a 20 fs timestep for 2.5 × 10^4^ steps, setting the pressure through isotropic coupling. Then, the simulation was run for 1.25 µs using a 20 fs time step for 6.25 × 10^7^ steps setting the pressure through semi‐isotropic coupling. The simulation and the two equilibration steps were performed at the NPT ensemble, setting the temperature at 310 K (to reproduce the conditions elsewhere^[^ ^43^ ^]^) by using the v‐rescale thermostat and 1 bar pressure using the Berendsen algorithm.

The output structures of the fibers and PM model individually after 1.25 µs of simulation (Figure 3A; Figure S4A, Supporting Information) were dried, removing the waters and ions from both systems. The fibers were inserted in PM box with dimensions in the *x*‐ and *y*‐axis 26.37 nm × 17.58 nm and the *z*‐axis were modified to 30 nm to fit the fibers in the box. The fibers were rotated 90° in the *y*‐axis to obtain an infinite interconnected fiber in the *x*‐direction, separated 7 nm from the center of the PM, and rotated 45° in the *x*‐direction (Figure 3B; Figure S4C, Supporting Information). A similar setup was used previously in Martini‐based simulations of actin fibers interacting with a lipid membrane.^[^ ^77^ ^]^ The systems were solvated with a 75% content of explicit Martini water, and a 25% content of explicit Martini anti‐freezing water particles and 100 mm NaCl. The system was minimized using the steepest decent minimization algorithm for 2 × 10^3^ kJ mol^−1^ nm^−1^ and the simulations were run for 1.25 µs using a 20 fs time step for 6.25 × 10^7^ steps using GROMACS (version 2022).^[^ ^69^ ^]^ The simulations were performed at the NPT ensemble, setting the temperature at 310 K by using the v‐rescale thermostat and 1 bar pressure through semi‐isotropic coupling using the Berendsen algorithm.

The energy analysis was performed using the energy function implemented in GROMACS (version 2021).^[^ ^72^ ^]^ The contributions of the Lennard–Jones and the Coulombic energies of the interactions between the lipids in the PM and the charged residues of the fibers (i.e., glutamic acids, lysines, or both in the case of **EK**) were summed. The average during the last 0.25 µs of the simulation was represented, normalized by the number of charges of each peptide (9 for **E9** and K9, 7 for **E7** and K7, 6 for **E6**, **K6**, and **EK**, and 3 for **E3** and **K3**). All the visualizations were also rendered using Chimera X 1.6.1.^[^ ^73^ ^]^

### Fiber Preparation

The lyophilized peptides were ordered from GenScript Biotech and Merk. Except for the FT‐IR experiments, the peptides were dissolved in miliQ water with the pH respectively adjusted to 5, 7, or 9 by dropwise addition of NaOH. The final concentration of the samples was 4 mm for all the peptides except for **E6** that was prepared at 2 mm given that at 4 mm it forms highly viscous gels difficult to pipette. The samples were sonicated for 10 min and annealed for 30 min at 80 °C and slowly cooled down in the bath until RT.

For the FT‐IR experiments, the samples were prepared in D_2_O at 10 mm instead of miliQ water, following the same protocol described before.

### Circular Dichroism

The secondary structure was measured using a Jasco J‐815 spectrophotometer. The temperature was maintained at RT and the sample was placed in a quartz cuvette with 0.1 cm of optical path. The CD spectra was recorded from 260 to 190 nm with a 0.2 nm data pitch with a 50 nm min^−1^ scanning speed and 5 accumulations. The DIT was set at 1 s and the bandwidth to 1 nm. The measurements were performed at different concentrations given the changes in the limit of detection when the fibers are not at their optimal pH. Most commonly, the samples were measured at 50 µm. However, at pH 7, **E6**, K3, K6, and **K7** were measured at 100 µm, and **K9** at 500 µm. The sample was placed in a quartz cuvette with an optical path length of 0.1 cm. The recorded CD signal was normalized to represent the data as molar ellipticity, being *c* the concentration (M) and *l* the optical path length (cm):

(1) $$\Delta E=100\frac{\mathrm{CD}\;\left(\mathrm{mdeg}\right)}{3300cl}\left(M^{1}cm^{-1}\right)$$

### Transmission Electron Microscopy (TEM)

An aliquot of 5 µL at 10 µm for each fiber was deposited on a 400 Mesh Copper (100) carbon films from EM Resolutions, dried for 5 min, and the solution was removed by capillary action using filter paper and dried for 10 min more. Then the samples were stained with 1.5% uranyl acetate for 5 min followed by 2 washes with miliQ water to homogenize the staining. The images were acquired with a JEOL JEM‐1400PLUS (40–120 kV, HC pole piece) LaB6‐TEM equipped with a GATAN US1000 CCD camera (2k – 2k).

### Rheological Assessment

The rheological properties of the supramolecular materials were evaluated using the Discovery HR‐2 controlled stress rheometer (TA instruments). A cone plate of 15 mm diameter with a 0.078 mm gap was used in all tests. The viscosity as a function of shear rate (0.1 to 1000 s^−1^) was conducted at a constant temperature of 37 °C. In all experiments, the sample was left to acquire the desired temperature for 1 min. Three tests were performed for each peptide, using 400 µL of sample at 100 µm concentration for each trial.

### Dynamic Light Scattering (DLS) Zeta Potential Analysis

Charges values were measured by using a DLS (Malvern Zetasizer). As zeta potential analysis is optimized for spherical particles, additional steps were taken to address the non‐spherical and polydisperse nature of the fibers. Specifically, the samples were subjected to brief sonication to fragment the long fibers into smaller, more diffuse structures prior to measurement. This approach minimized the effects of fiber–fiber interactions and sample heterogeneity, enabling a qualitative comparison of zeta potential values across the peptide library. Measurements were carried out under identical conditions for all samples. Thus, after annealing, the samples containing the biomaterial dissolved in Milli‐Q water at a 100 µm concentration were sonicated for 5 min right before taking the measurements in order for the machine to detect all the charges of the structures. A volume of 600 µL of each sample was used to measure each parameter on 3 tests.

### Fourier‐Transform Infrared (FT‐IR)

The data was recorded using an Invenio‐X Bruker FT‐IR. The samples were placed between two CaF_2_ windows of 32 mm, separated by a Teflon spacer of 50 µm. The measurements were recorded with a 1 cm^−1^ resolution and 25 accumulations. The relative absorptions of D_2_O and background were subtracted to the raw data and normalized by the value of the hydrogen bonding signal to compare the samples.

### Small‐Angle X‐Ray Scattering (SAXS)

The experiments were performed using a Rigaku 3‐pinhole PSAXS‐L equipment, operating at 45 kV and 0.88 mA. The equipment includes a MicroMax‐002+ X‐ray generator, consisting of a microfocus sealed tube source and an integrated Cu Kα X‐ray generator unit (photons wavelength *λ* = 1.5406 Å). Both the flight path and sample chamber are maintained under vacuum conditions. The scattered X‐rays are detected by a Dectris hybrid photon counting detector (Dectris EIGER2 R 1M‐RW), which provides micrometric spatial resolution (75 µm × 75 µm) over an active area of 77.1 mm × 79.65 mm with 1 m pixels. Azimuthally averaged scattered intensities were obtained as a function of the scattering vector *q* = 4*πλ* – 1 sin(*θ*/2), where *θ* is the scattering angle. Reciprocal space calibration was carried out using silver behenate as the standard. The samples were measured in transmission geometry with a sample‐to‐detector distance of 2 m, covering a *q*‐range of 0.008 to 0.2 Å^−1^ by combining 9 different detector positions with a 3⋅3 Detector Scan Profile (DSP). Seven consecutive measurements were taken, with an exposure time of 1000 s per scan. Experiments were performed at room temperature, using boron‐rich capillaries with a 2 mm thickness. For each sample, the solvent background was measured in the same capillary and with the same DSP and properly subtracted from the total signal. All the **E9**, **E6**, and **E3** fibers were first annealed in water at pH 5 at a concentration of 10 mm and then diluted in water or in cell media reaching a final concentration of 1 mm for the respective measurements. SAXS results for **E9**, **E6**, and **E3** fibers were analyzed using the SasView software,^[^ ^78^ ^]^ employing a cylinder model^[^ ^79^, ^80^, ^81^ ^]^ with Gaussian distributions for fiber radius *R* and length *L*. Figure S5A–C (Supporting Information) presents the SAXS data with the corresponding fit of the cylinder model to the experimental data in water at pH 5 and in cell media. Table S2 (Supporting Information) summarizes the values obtained for the parameters of the cylinder model, where Ro (nm) represents the expected radius of the cylinder, Lo (nm) its expected length, both accompanied by their respective variance σ. Figure S5D,E (Supporting Information) shows the distribution functions corresponding to the results in water at pH 5.

### Cell Culture

Human neural progenitor cells (hNPCs) were differentiated from human embryonic cell line HUES‐64 (Harvard University), following Brennand laboratory's protocol.^[^ ^82^ ^]^ Obtained hNPCs were cultured on laminin coated glass coverslips as previously described.^[^ ^12^ ^]^ Briefly, 12 mm glass coverslips (Fisher Scientific) were coated with poly‐D‐Lysine (PDL, 0.01 mg mL^−1^ in water, Sigma‐Aldrich) for 3 h at 37 °C. The plates were then rinsed with Milli‐Q water three times and allowed to dry for 4 h. After washing the PDL coatings, Laminin (10 µg mL^−1^, Thermo Fisher) was incubated for 1 h at 37 °C. Cells at a density of 50 000 cells per well in 24 well plates were seeded after aspirating the laminin solution. Cells were maintained in hNPC‐media composed of DMEM/F12 (Life Technologies) supplemented with 1× N‐2 supplement (Life Technologies), 1× B27‐RA (Life Technologies), 20 ng mL^−1^ FGF2 (Life Technologies), and 1% Pen/Strep (Gibco), with media changes every second day.

### Cell Treatment with Peptide Fibers

Under sterile conditions, 100 µm of peptide solution was prepared by mixing an annealed 1 mm peptide stock solution with the hNPC‐media.

### Cell Viability Assay

Cell viability was assessed via a live/dead assay after incubating the cells for 1 week in vitro. Briefly, cells were washed phosphate‐buffered saline (PBS, Gibco) followed by incubation in PBS containing 8 µm calcein acetoxymethyl (calcein AM, Invitrogen) and 1 µg mL^−1^ ethidium homodimer‐1 (EthD‐1, Sigma‐Aldrich) for 15 min. Samples were washed in PBS before imaging with a Zeiss LSM800 laser scanning confocal microscope excited at 494 and 535 nm and read at 517 and 617 nm.

### Western Blot Analysis

Following 24 h of incubation, cells were mechanically disrupted using a cell scraper, and protein was extracted in Pierce RIPA buffer (Thermo Scientific) with a Halt protease and phosphatase inhibitor cocktail (Thermo Scientific). Extracted protein was horn sonicated prior to quantification using a Pierce BCA Protein Assay Kit (Thermo Scientific). For WB analysis lysates were sonicated and protein extracts were separated by SDS‐PAGE followed by electro transfer to a nitrocellulose membrane (Bio‐Rad). The membranes were blocked in Tris‐buffered saline (TBS, Bio‐Rad) + 5% non‐fat dry milk (Bio‐Rad) and then incubated overnight at 4 °C with primary antibodies. Primary antibodies (ITGB1 (Sigma‐Aldrich, MAB2079Z), TUJ1 (abcam, ab78078), CASP3 (Cell Signaling, 14220), cCASP3 (Cell Signaling, 9664), ACTIN (Cell Signaling, 8H10D10)) were diluted in TBS + 0.1% Tween + 5% BSA (Sigma Aldrich). After several washes in TBS + 0.1% Tween, membranes were incubated with their corresponding secondary HRP‐conjugated antibodies (Thermo Fisher). A radiance bioluminescent ECL substrate kits (Pierce ECL Western Blotting Substrate, Thermo Fisher) was used to detect protein signals through Image Quant LAS4000 mini‐imager (General Electric) on the automatic setting.

### Proteomic Analysis

Samples of 3 independent replicates for each condition (Control, **E9**, **E6**, and **E3**) were analyzed by nanoLC‐MS/MS. A total of 10 µg of each sample were digested with trypsin/LysC and cleaned up using the iST PreOmics Kit. Those samples were then analyzed by nanoLC‐MS/MS using a Data Independent Acquisition (DIA), library‐free, label‐free strategy. Before proceeding to the differential abundance analysis, a quality control analysis (QC) was performed to give an insight on the overall technical quality of the data. This involved working just with those protein groups with quantification values in all runs (full observations) and using the locally estimated scatterplot smoothing normalization (LOESS) as the normalization strategy. These two actions ensure removal of any biases when comparing conditions with different identification numbers, and therefore, allow to proceed to the differential abundance analysis. MaxLFQ from DIA‐NN was used for protein group quantification and applied standard cutoffs for the fold change (|FC| > 1.5) and adjusted *p*‐value (*p* < 0.05) to define significant protein groups in all comparisons. Gene Ontology (GO) analysis was performed using the online tools Metascape and DAVID.^[^ ^83^, ^84^, ^85^, ^86^, ^87^ ^]^

### Immunocytochemistry

For immunofluorescence, hNPCs were fixed in 4% paraformaldehyde at RT for 30 min., blocked with PBS + 0.1% Triton X‐100 + 1–5% Normal Horse Serum and incubated with primary antibodies (ab78078 and ab105389) ON at 4 °C. The next day, three rinses with PBS + 0.1% Triton X‐100 + 1–5% Normal Horse Serum were performed, followed by an incubation with the appropriate anti‐rabbit Alexa 555 (A31572), or anti‐mouse Alexa 488 (A21202). DAPI (Molecular Probes D3571 at 1:1000) was used to stain nuclei. Finally, the preparations were cover‐slipped with Immu‐Mount (Thermo Fisher) for imaging.

### Image Acquisition and Analysis

Fluorescent preparations were viewed and captured with a Zeiss LSM800 laser scanning confocal microscope. Images were processed with ImageJ and assembled with Adobe Photoshop 2024 version 24.7 with adjustments for contrast, brightness and color balance to obtain optimum visual reproduction of data. For quantification of fluorescently stained images, a minimum of 20 randomly selected images was included for each condition.

### Statistical Analysis

Statistical analysis in Figures 1, 2, and 3D was performed using PyCharm software. The data was reported as mean ± SD. For computational simulations the analysis was calculated for a single simulation (*n* = 1). For the experimental analysis of Z‐potential and rheology in Figure 2C and D the mean was obtained from the data obtained from three different measurements (*n* = 3). Statistical analysis in Figures 3E and 4 was performed using GraphPad Prism10 software. All data are reported as mean ± SEM. For each statistical analysis, the datasets were first evaluated for fit into a Gaussian distribution using the D'Agostino–Pearson omnibus normality test. To compare ≥3 experimental conditions, one‐way analysis of variance (ANOVA) followed by a Tukey post hoc test (parametric), or Kruskal–Wallis one‐way analysis of variance (non‐parametric) was performed.

## Conflict of Interest

The authors declare no conflict of interest.

## Supporting information



Supporting Information

## Data Availability

The data that support the findings of this study are available from the corresponding author upon reasonable request.
