# Supplementary material for: Peptide Electrostatic Modulation Directs Human Neural Cell Fate
Source: Adv Sci (Weinh). 2025 Sep 29;13(2):e07946. doi: 10.1002/advs.202507946 (PMC12786318; doi:10.1002/advs.202507946)
Supplement: Supplementary file 1 — Supporting Information [file ADVS-13-e07946-s001.pdf]

Supporting Information

Peptide Electrostatic Modulation Directs Human Neural Cell Fate

Laura Perez-Chirinos,[#] Xavier Barceló,[#] M. Gabriella Chiariello, Irene Sanz, Amaia Iturrospe, Arantxa Arbe, Juan Alberto Ortega, Siewert J. Marrink, Aitziber L. Cortajarena,* Zaida Alvarez,* Ivan R. Sasselli.*

E-mail: i.sasselli@csic.es ; zalvarez@ibecbarcelona.eu ; alcortajarena@cicbiomagune.es

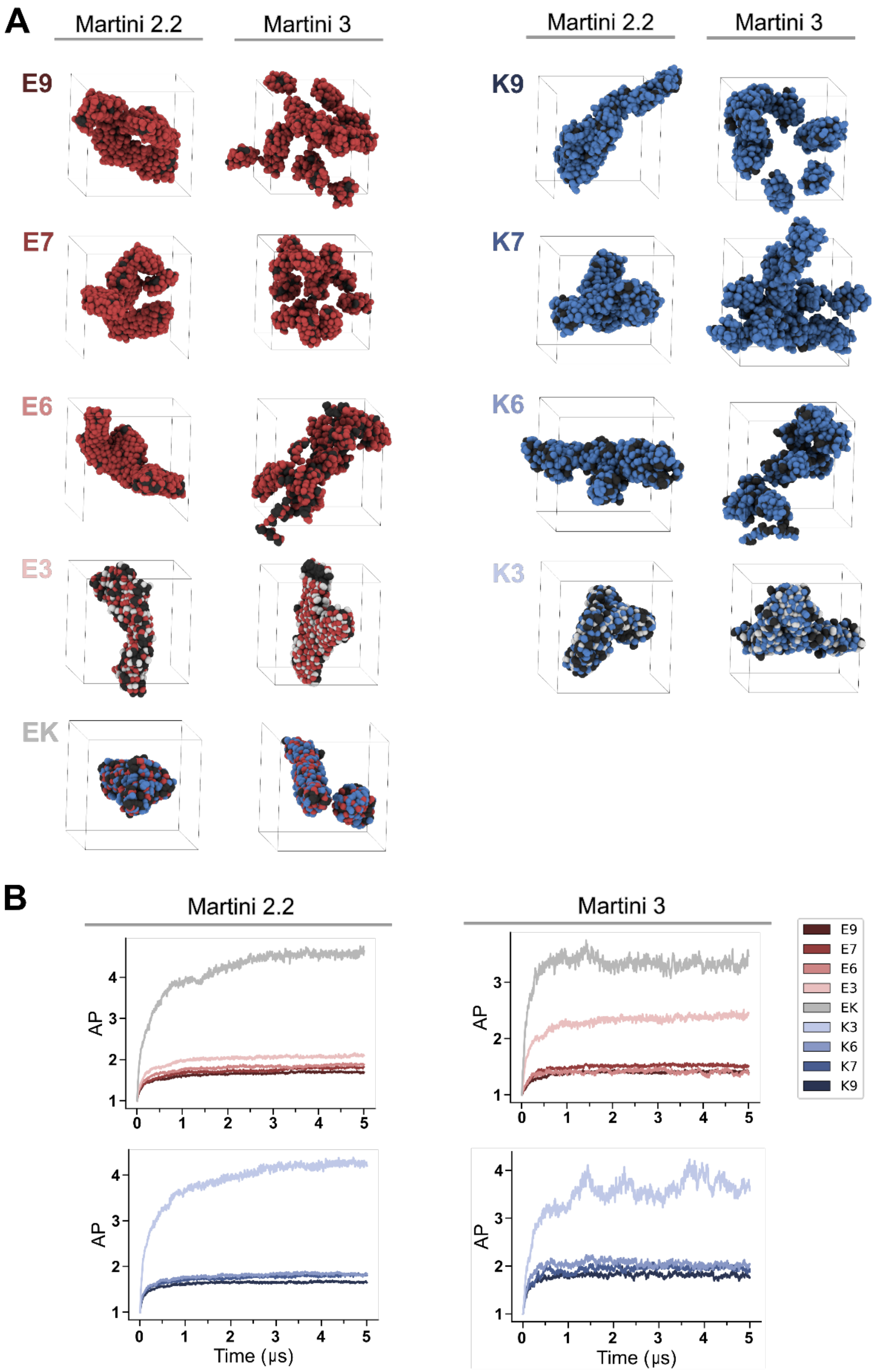

Figure S1. Simulations of the nine peptides from the library using Martini 2.2 and Martini 3 forcefields (A) Snapshots of the final simulation frame. (B) AP during the 5 μs simulation time.

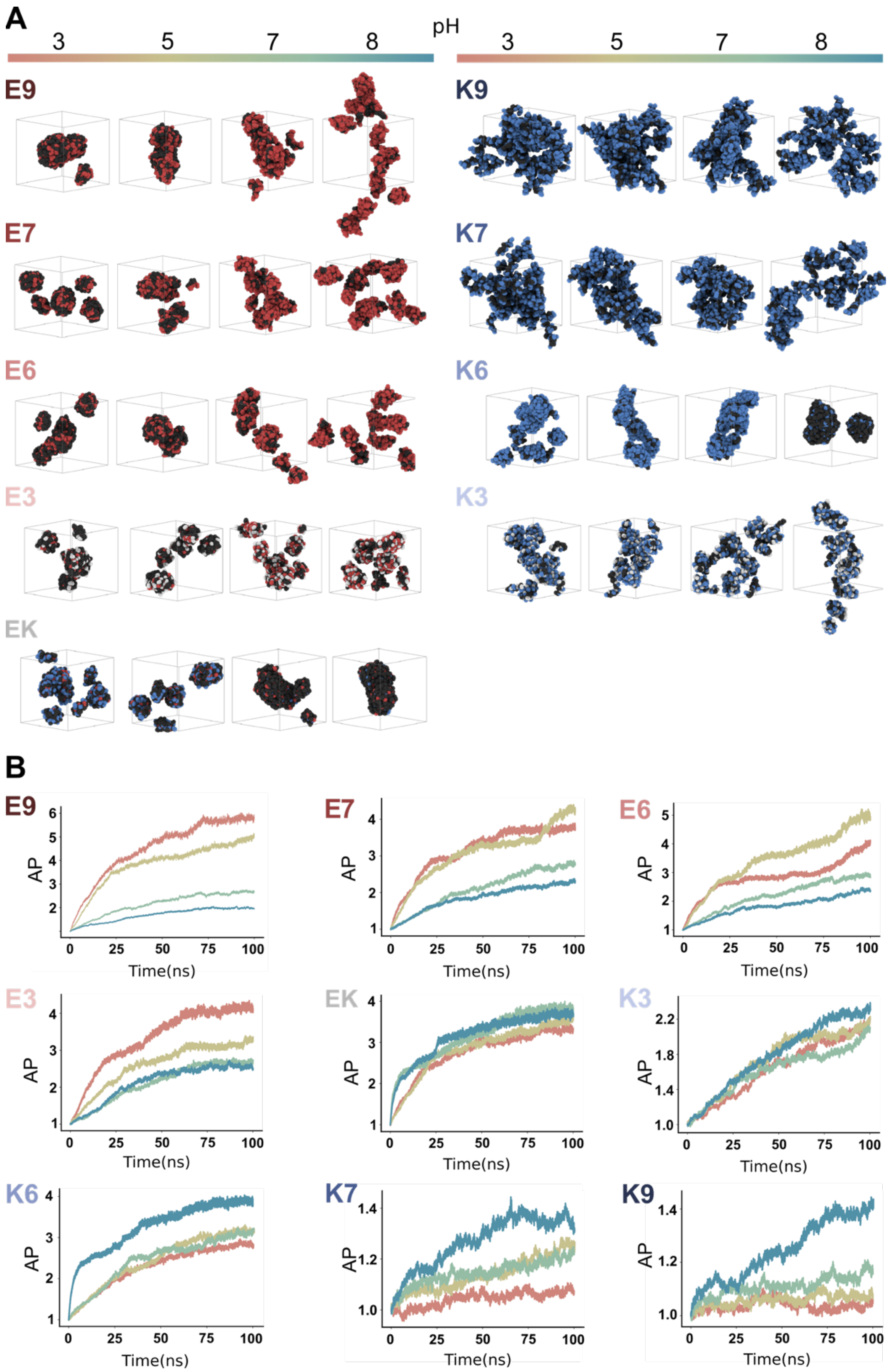

Figure S2. (A). Last simulation frame of the nine peptides from the library with the titratable model at the varying pH values (3, 5, 7, and 8). (B). AP during the 100 ns simulation time of the peptides at pH 3 (red), 5 (yellow), 7 (green), and 9 (blue).

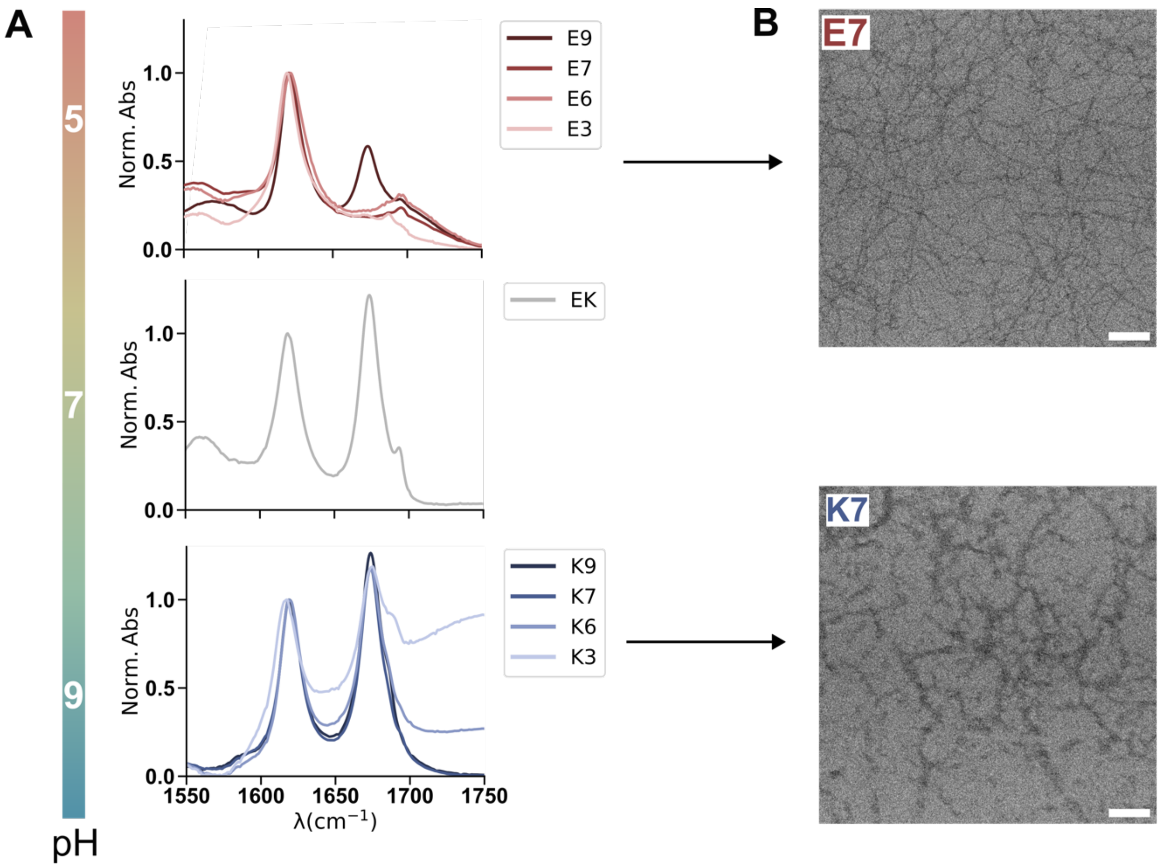

Figure S3. (A) FT-IR spectra of the supramolecular fibers of the peptide library self-assembled at their optimal pH. **E9**, **E7**, **E6**, and **E3** at pH 5. **EK** at pH 7. **K9**, **K7**, **K6**, and **K3** at pH9. (B) TEM micrographs of the fibers **E7** at pH 5 and **K7** at pH 9. Scale bars: 100 nm.

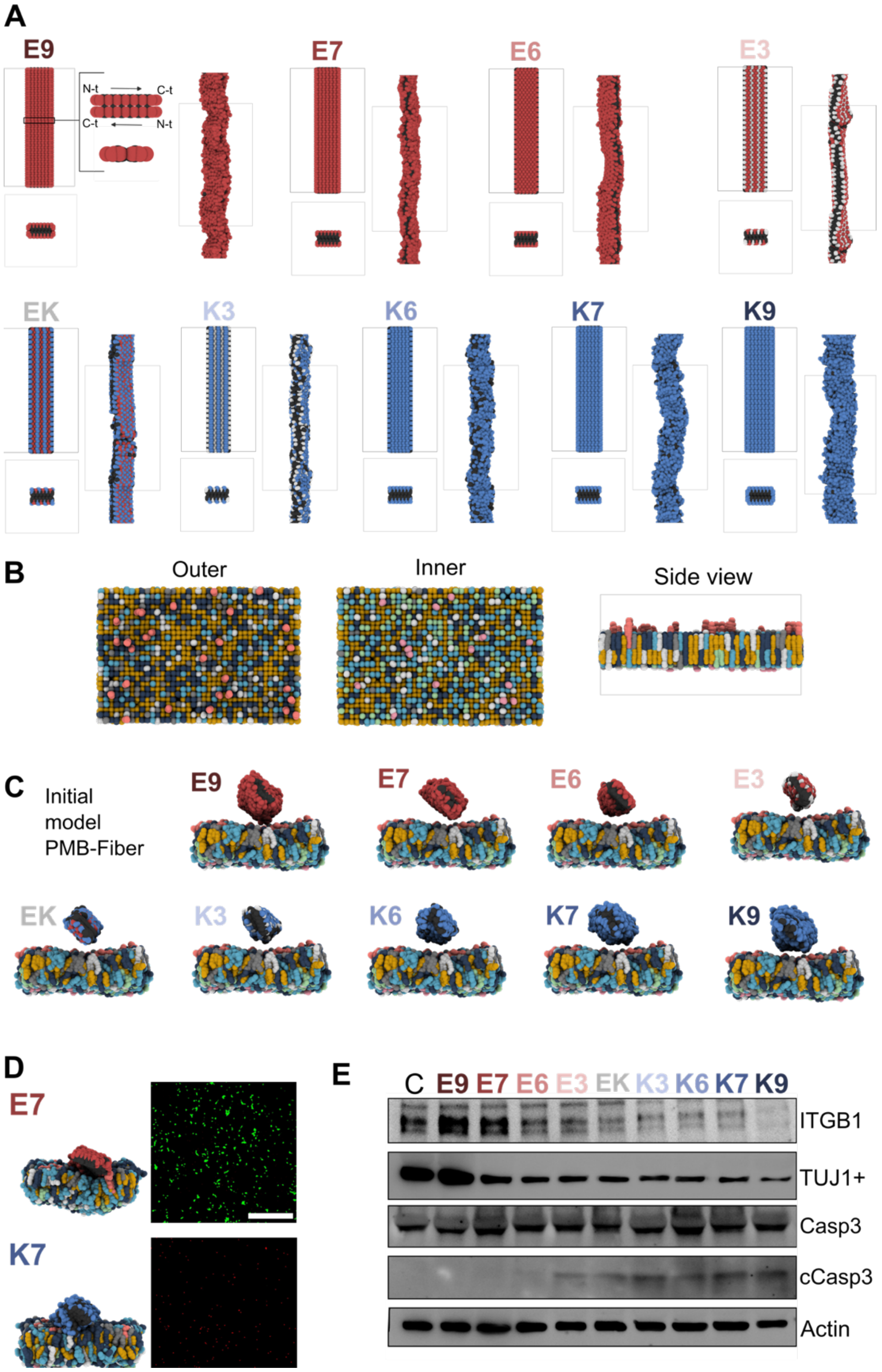

Figure S4. Fiber-PM interaction. (A) Initial structural models (left) and snapshot of the simulation after 1.25 µs (right) of the nine fibers in the peptide library. (B) Initial model of the plasma membrane (PM), illustrating the organization of the outer and inner leaflets. (C) Initial model of the fiber-PM system for all the fibers. (D) Final structures of **E7** and **K7** fibers after 5 µs of molecular dynamics simulation (left). Representative confocal micrographs of hNPCs treated with **E7** and **K7** fibers, stained with calcein (green) to indicate live cells and propidium iodide (red) to indicate dead cells (right). Scale bar corresponds to 100 µm. (E) Western Blot

(WB) for ITGB1, TUJ1+, Casp3 (Caspase-3), cCasp3 (Cleaved Caspase-3), and Actin for the different fibers and the control (C).

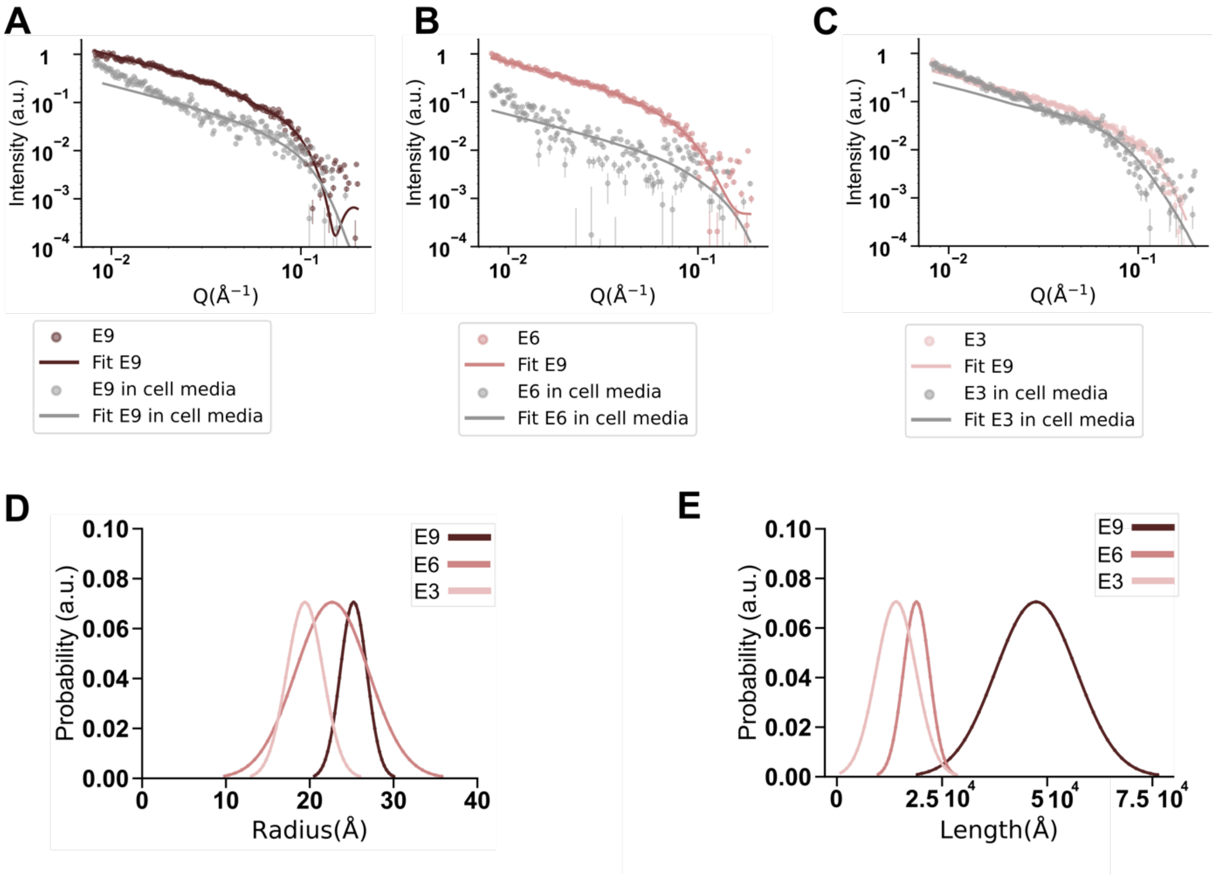

Figure S5. (A, B, C). SAXS data of the (A) **E9**. (B) **E6**. (C) **E3** fibers in water at pH 5 and in cell media. Dots: experimental data from the average of seven different measurements (n = 7). Lines: fit of the cylinder model to the experimental data. Further details in experimental section. (D, E) Distribution functions deduced from the fit of the cylinder model to the SAXS results of water at pH 5. (D): cylinder lengths (E): cylinder radii. The functions are normalized to have the same value at the maxima.

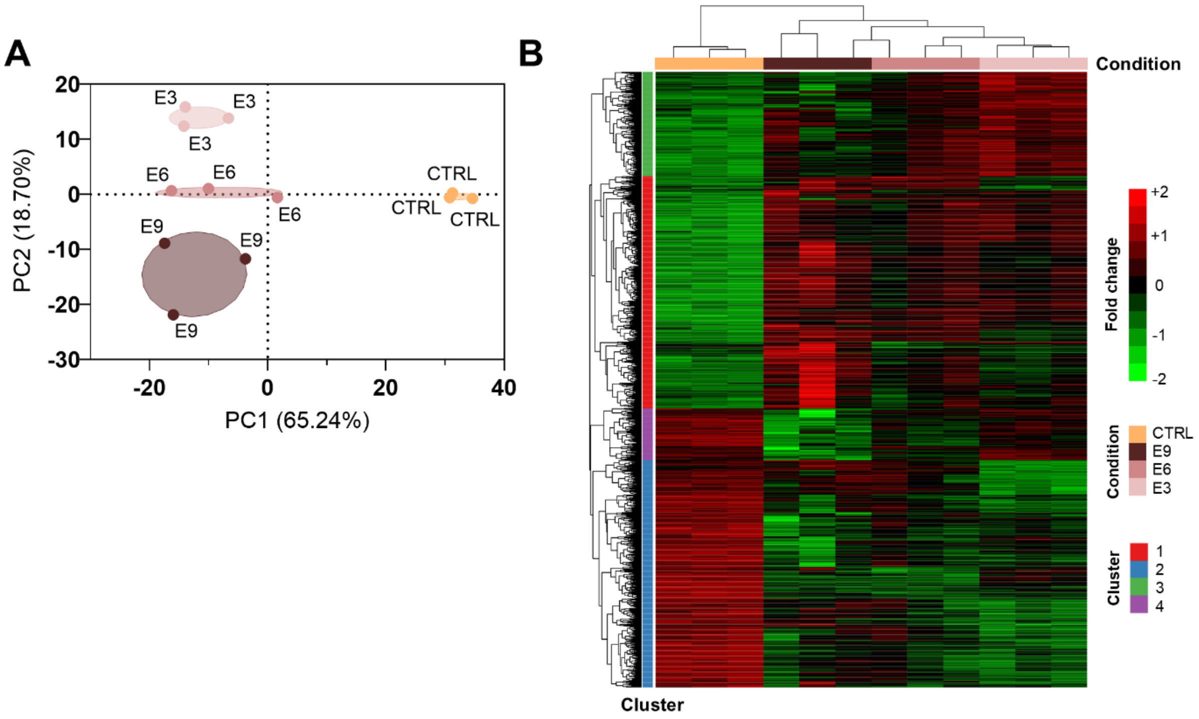

Figure S6. Differential electrostatic charges in self-assembled fibers drive distinct neural cell fate outcomes in human neural progenitor cells (hNPCs). (A) Principal Component Analysis (PCA) of the proteomic dataset (3,690 proteins; n = 3) showing distinct clustering of experimental groups (**E3**, **E6**, **E9**) and controls. (B) Hierarchical clustering heatmap of differentially abundant proteins, identifying four distinct protein clusters.

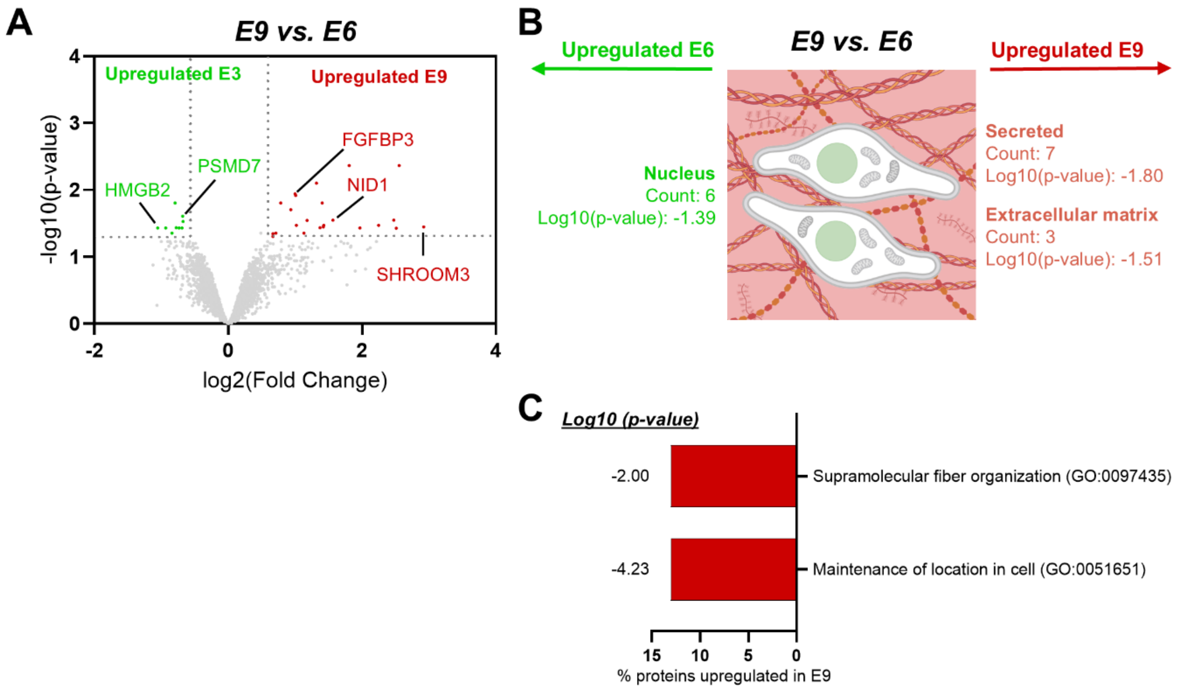

Figure S7. Differential electrostatic charges in self-assembled fibers influence neural cell fate in hNPCs. (A) Volcano plot showing fold-change (x-axis) versus significance (-log10 p-value, y-axis) for protein expression in hNPCs treated with **E6** or **E9** fibers. Upregulated proteins ($|FC| > 1.5$ and $p < 0.05$) are marked in red (**E9**) or green (**E6**), with key proteins labeled. (B) Schematic representation of the subcellular localization of significantly upregulated proteins ($p < 0.05$) in **E6** (green) and **E9** (red) treatment groups. (C) Gene Ontology (GO) analysis highlighting enriched biological processes, with p-values and percentages of total protein content displayed.

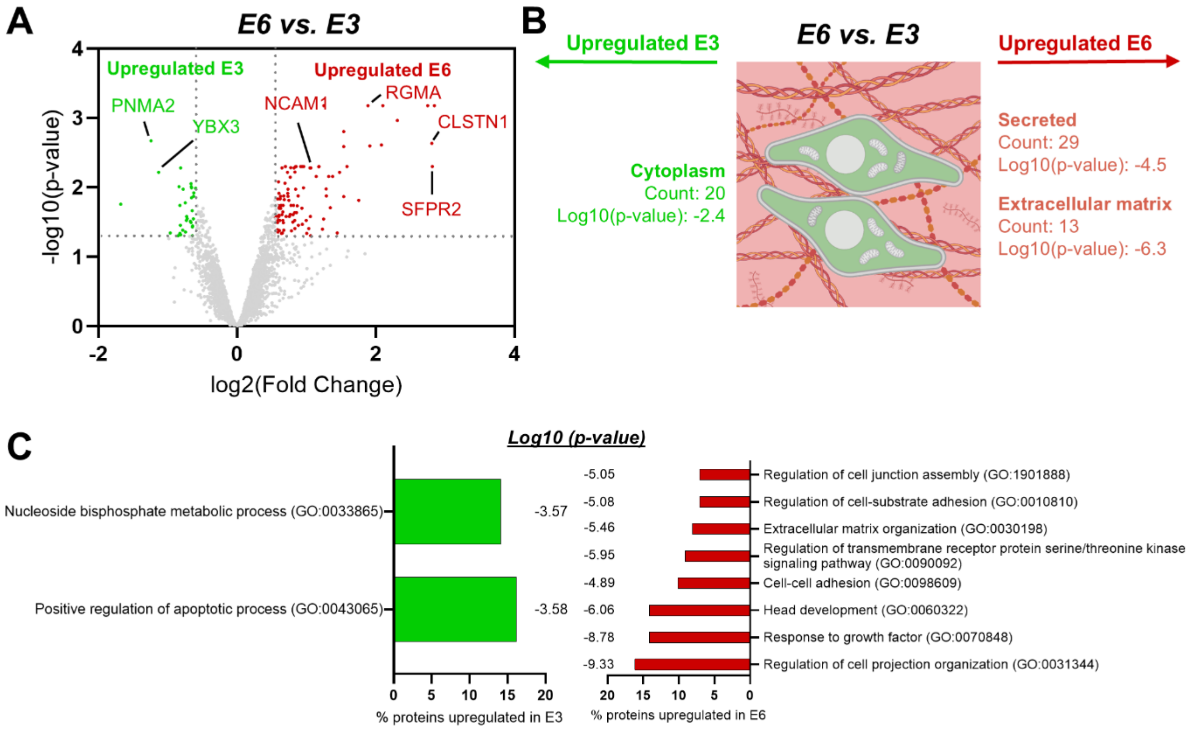

Figure S8. (A) Volcano plot showing fold-change (x-axis) versus significance (-log10 p-value, y-axis) for protein expression in hNPCs treated with **E3** or **E6** fibers. Upregulated proteins ($|FC| > 1.5$ and $p < 0.05$) are marked in red (**E6**) or green (**E3**), with key proteins labeled. (B) Schematic representation of the subcellular localization of significantly upregulated proteins ($p < 0.05$) in **E3** (green) and **E6** (red) treatment groups. (C) Gene Ontology (GO) analysis highlighting enriched biological processes, with p-values and percentages of total protein content displayed.

Table S1. Abundance of the lipids in the inner/outer leaflets of the plasma membrane. The total number and the percentage composition of the lipids within the membrane is represented. The names of the lipids correspond the abbreviations used in the Martini model. The percentage was rounded to two decimals.

Lipid	Outer membrane		Inner membrane	
	Total	Percentage	Total	Percentage
**Phosphatidylcholine (PC)**				
DPPX	53	5.26	30	2.98
POPX	88	8.74	49	4.87
DOPC	22	2.18	12	1.19
PFPC	6	0.59	3	0.30
PAPC	46	4.57	26	2.58
PUPC	17	1.69	10	0.99
OIPC	6	0.59	3	0.30
OUPC	4	0.40	2	0.20
**Phosphatidylethanolamine (PE)**				
POPE	13	1.29	25	2.48
PAPE	31	3.08	61	6.06
PUPE	50	4.96	98	9.73
OIPE	1	0.10	3	0.30
OAPE	7	0.69	13	1.29
OUPE	7	0.69	14	1.39
**Sphingomyelin (SM)**				
DPSM	58	5.76	15	1.49
POSM	7	0.69	2	0.20
PNSM	13	1.29	3	0.30
PBSM	11	1.09	3	0.30
**Phosphatidylserine (PS)**				
DPPS	0	0.00	5	0.50
POPS	0	0.00	25	2.48
PAPS	0	0.00	28	2.78
PUPS	0	0.00	34	3.38

OUPS	0	0.00	7	0.69
**Phosphatidylinositol (PI)**				
POPI	0	0.00	13	1.29
PIPI	0	0.00	5	0.50
PAPI	0	0.00	13	1.29
PUPI	0	0.00	20	1.99
**Phosphatic acid (PA)**				
POPA	0	0.00	1	0.10
PAPA	0	0.00	3	0.30
**Phosphatidylinositol phosphates (PIPs)**				
POP1	0	0.00	2	0.20
PAP1	0	0.00	3	0.30
POP2	0	0.00	2	0.20
PAP2	0	0.00	3	0.30
POP3	0	0.00	2	0.20
PAP3	0	0.00	3	0.30
**Ceramide (CER)**				
DPCE	0	0.00	4	0.40
DBCE	0	0.00	1	0.10
DPCE	4	0.40	0	0.00
DBCE	1	0.10	0	0.00
PNCE	1	0.10	1	0.10
POCE	0	0.00	0	0.00
**Glycolipid (GM1)**				
DPG1	9	0.89	0	0.00
DBG1	2	0.20	0	0.00
POG1	1	0.10	0	0.00
PNG1	2	0.20	0	0.00
**Glycolipid (GM3)**				
DPG3	9	0.89	0	0.00
DBG3	2	0.20	0	0.00
POG3	1	0.10	0	0.00

PNG3	2	0.20	0	0.00
**Cerebrosides**				
DPGS	49	4.86	0	0.00
DBGS	9	0.89	0	0.00
POGS	6	0.59	0	0.00
PNGS	11	1.09	0	0.00
**Lysophosphatidylcholine (LPC)**				
PPC	2	0.20	1	0.10
IPC	1	0.10	1	0.10
**Lysophosphatidylethanolamine (LPE)**				
PPE	1	0.10	1	0.10
IPE	1	0.10	2	0.20
**Diacylglycerol (DAG)**				
PODG	1	0.10	1	0.20
PADG	3	0.30	3	0.30
**Cholesterol (CHOL)**				
CHOL	449	44.59	451	44.79

Table S2. Values obtained for the parameters of the cylinder model, where R_0 (nm) represents the expected radius of the cylinder, L_0 (nm) its expected length, both accompanied by their respective variance σ.

Fiber	R_0 (nm)	σ (nm)	L_0 (nm)	σ (nm)
**E3** in water	1.94	0.217	1408	477
**E3** in cell media	1.49	0.387	8659	3963
**E6** in water	2.27	0.464	1890	311
**E6** in cell media	1.62	0.234	1962	728
**E9** in water	2.52	0.159	4735	962
**E9** in cell media	2.00	0.162	1522	339
